# Proteolysis triggers self-assembly and unmasks innate immune function of a human α-defensin peptide†
†Electronic supplementary information (ESI) available: Tables of amino acid sequences and characterization of peptides employed in this work, calculated sedimentation coefficients, sedimentation velocity results, and sedimentation equilibrium results for proHD6. Figures of characterization of HD6 in ileal fluid, working model of HD6 maturation, SDS-PAGE, HPLC traces, antibacterial activity assays and *in vitro* trypsin-catalyzed degradation of recombinant proHD6, characterization of products from trypsinized proHD6, TEM of trypsinized proHD6 in Tris-maleate buffer and at different time points, SEM of bacterial agglutination by trypsinized proHD6, cytotoxicity studies of proHD6 and HD6 against T84 cells, sedimentation velocity analysis, and sedimentation equilibrium analysis of proHD6. See DOI: 10.1039/c5sc04194e



**DOI:** 10.1039/c5sc04194e

**Published:** 2015-12-10

**Authors:** Phoom Chairatana, Hiutung Chu, Patricia A. Castillo, Bo Shen, Charles L. Bevins, Elizabeth M. Nolan

**Affiliations:** a Department of Chemistry , Massachusetts Institute of Technology , Cambridge , MA 02139 , USA . Email: lnolan@mit.edu ; Fax: +1-617-324-0505 ; Tel: +1-617-452-2495; b Department of Microbiology and Immunology , University of California Davis School of Medicine , Davis , CA 95616 , USA; c Department of General Internal Medicine and Gastroenterology and Hepatology , The Cleveland Clinic Foundation , Cleveland , OH 44195 , USA

## Abstract

Human α-defensin 6 (HD6) is a unique peptide of the defensin family that provides innate immunity in the intestine by self-assembling to form higher-order oligomers that entrap bacteria and prevent host cell invasion. Here, we report critical steps in the self-assembly pathway of HD6. We demonstrate that HD6 is localized in secretory granules of small intestinal Paneth cells. HD6 is stored in these granules as an 81-residue propeptide (proHD6), and is recovered from ileal lumen as a 32-residue mature peptide. The propeptide neither forms higher-order oligomers, nor agglutinates bacteria, nor prevents *Listeria monocytogenes* invasion into epithelial cells. The Paneth cell granules also contain the protease trypsin, and trypsin-catalyzed hydrolysis of proHD6 liberates mature HD6, unmasking its latent activities. This work illustrates a remarkable example of how nature utilizes a propeptide strategy to spatially and temporally control peptide self-assembly, and thereby initiates innate immune function in the human intestine.

## Abstract 2

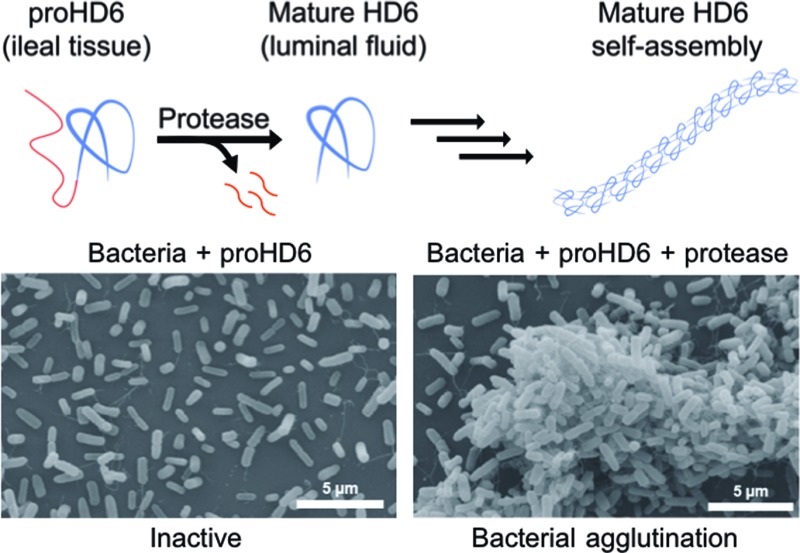
Human α-defensin 6 (HD6) is a unique peptide of the defensin family that provides innate immunity in the intestine by self-assembling to form higher-order oligomers that entrap bacteria and prevent host cell invasion.

## Introduction

The innate immune system mediates homeostasis at mucosal surfaces, in part by providing protection from microbial invasion. Host-defense peptides are abundant and important players in the interplay between host and microbe at mucosal surfaces.^1,2^ The intestine harbors the largest reservoir of colonizing microbes, termed the microbiota, and this diverse community is mostly comprised of microorganisms that are beneficial to the host. Nevertheless, some resident members of this community, as well as many transient microbes, can invade the epithelium and thus pose a significant challenge for the immune system to effectively protect the host and maintain intestinal homeostasis. Paneth cells, secretory cells located at the bases of the crypts of Lieberkühn in the small intestine, contribute to mucosal innate immunity by releasing a cocktail of host-defense peptides and proteins in response to microbial stimuli.^3–5^ In humans, Paneth cells express two α-defensins, human defensins 5 and 6 (HD5 and HD6).^6–9^ Defensins are small (2–5 kDa) cysteine-rich host-defense peptides expressed by epithelial cells and neutrophils. These peptides typically exhibit broad-spectrum antimicrobial activity.^2,10,11^ α-Defensins exhibit three regiospecific disulfide bonds (Cys^I^–Cys^VI^, Cys^II^–Cys^IV^, Cys^III^–Cys^V^) in the oxidized form, which stabilize a three-stranded β-sheet fold and confer protease resistance.^12–14^ The oxidized form of HD6, in contrast to HD5 and other characterized α-defensins, exhibits negligible *in vitro* antimicrobial activity.^12,15–18^ Based on *in vivo* model studies and *in vitro* characterization, HD6 operates by an unprecedented host-defense mechanism involving its unusual self-assembly properties.^15,17^ HD6 monomers oligomerize into extended structures termed “nanonets” and thereby entrap bacteria in the small intestinal lumen. This capture mechanism prevents bacterial invasion into host epithelial cells and subsequent dissemination to other organs.^15,17^ The HD6 nanonets have been observed *in vivo* and *in vitro*, with the former studies employing HD6 transgenic mice infected with the enteric pathogen *Salmonella enterica* serovar Typhimurium (*S*. Typhimurium).^17^

A number of fundamental chemical and biological questions about HD6 arise from prior studies. First, our understanding of HD6 is limited because the peptide has not been isolated and characterized from human intestine. Current assumptions about mature HD6 are based on one study in which the peptide was detected in urine specimens obtained from bladder cancer patients with surgically created ileal neobladders.^19^ There are also uncertainties related to HD6 storage and maturation. In particular, how do Paneth cells package and deploy a self-assembling peptide from granules, and how is formation of nanonets regulated? Analysis of human mRNA indicated that HD6 is translated as a 100-residue prepropeptide.^7^ This prepropeptide is predicted to contain a 19-residue N-terminal signal sequence that targets the peptide to the secretory pathway, and an 81-residue C-terminal region that corresponds to a putative mature HD6 and an intervening acidic propeptide domain (Table S1†).^7^ The HD6 propeptide has not been detected in a human specimen or characterized to date. Based on prior studies of α-defensins in humans^20–24^ and mice,^25,26^ whether HD6 is stored as mature peptide or as a propeptide is unclear because both cases are observed for other human α-defensins, and mice store α-defensins in their Paneth cells as mature peptides.

**Fig. 1:**
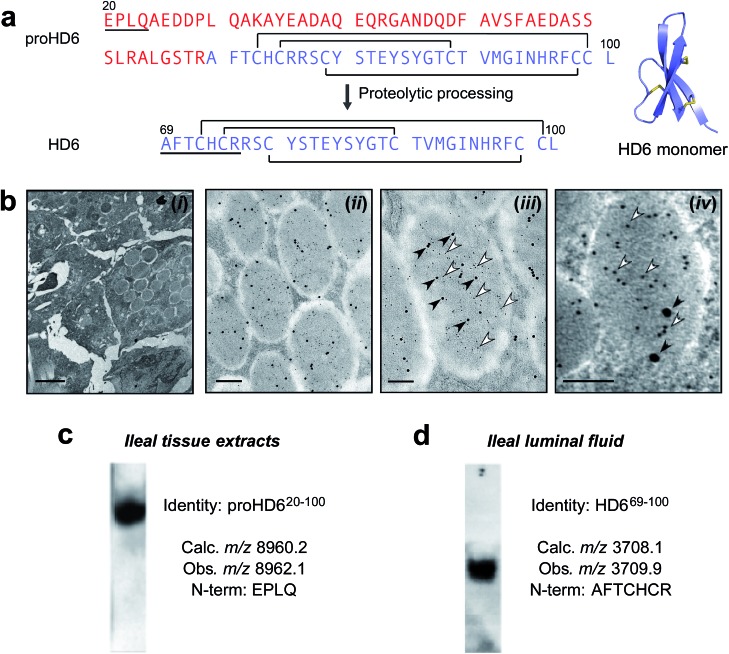
Identification and characterization of HD6 in intestinal tissue and fluid. (a) Left: primary amino acid sequences of proHD6 and HD6. The pro region is in red, the sequence of mature HD6 is in purple, and the disulfide linkages are depicted as black lines. The underlined amino acid sequences are matched with the results from Edman degradation shown in (c) and (d). Right: the crystal structure of mature HD6 (PDB ID: 1ZMQ).^12^ The disulfide bonds are shown in yellow. (b) Immunogold labeling transmission electron microscopy of human small intestinal tissue. (i) Left panel: low magnification of Paneth cell granules as a negative control where primary antisera was omitted, scale bar = 2 μm. (ii) Center-left panel: immunogold double-labeling of Paneth cell granules for HD5 (5 nm gold particles) and HD6 (15 nm gold particles), scale bar = 500 nm, (iii) center-right panel: immunogold labeling of a single Paneth cell granule demonstrating co-packaging of HD5 (5 nm gold particles, open arrowheads) and HD6 (15 nm gold particles, filled arrowheads), scale bar = 200 nm. (iv) Right panel: immunogold double-labeling of Paneth cell granules for HD5 (5 nm gold particle, open arrowheads) and trypsin (15 nm gold particles, filled arrowheads), scale bar = 100 nm. (c) and (d) Analysis of HD6 in human small intestinal tissue (c) and luminal fluid (d) by Western blot, mass spectrometry, and N-terminal Edman degradation. Tissue extracts of human ileum and ileal fluid aspirates were resolved by AU-PAGE, transferred onto a PVDF membrane, and probed for HD6. Two separate gels with mobility normalized according to migration of tracking dye to gel bottom are shown and reveal HD6 immunoreactivity in both samples.

Guided by the biophysical properties of HD6,^15^ the oxidatively folded regioisomer with Cys^I^–Cys^VI^, Cys^II^–Cys^IV^, Cys^III^–Cys^V^ bonds that is the focus of the current work, we reasoned that the quaternary structure of a proHD6 and mature HD6 differ. The HD6 crystal structure reveals that mature 32-residue HD6 monomers are arranged as a chain of tetramers where the N- and C-termini from four monomers form a hydrophobic pocket.^12,15^ On the basis of this structure^12^ and our studies of HD6 variants that have defective self-assembly properties,^15^ we hypothesized that the N-terminal pro sequence of proHD6 would interrupt the alignment of HD6 monomers and prevent the formation of higher-order oligomers. We therefore hypothesized that storage of HD6 as the propeptide in Paneth cells would prevent HD6 nanonets from forming in the granules. Moreover, the amino acid sequence of proHD6 (Fig. 1a) reveals that Arg^68^ provides a potential trypsin cleavage site, which is likely relevant because trypsin is expressed and released by human Paneth cells.^24,27^ Taken together, these observations provided us with a testable model whereby Paneth cells would package HD6 as an inactive propeptide, and that proteolytic processing by trypsin unleashes mature HD6 in the intestinal lumen.

Herein, we combine *ex vivo* analyses of human intestinal specimens with biophysical characterization and *in vitro* functional studies of HD6 and its propeptide to interrogate this model and characterize the HD6 maturation pathway. We report, for the first time, the detection and analysis of HD6 from samples of human intestinal tissue and luminal fluid. We demonstrate that an 81-residue proHD6 isoform exhibiting an N-terminal extension is found in ileal tissue and 32-residue mature HD6 is found in luminal fluid. We establish that proHD6 is an inactive isoform. The N-terminal region of proHD6 suppresses self-assembly and renders proHD6 unable to agglutinate bacteria and protect human epithelial cells from bacterial invasion. Moreover, we show that proHD6 is a substrate for trypsin, and that trypsin-catalyzed hydrolysis of proHD6 yields the 32-residue mature HD6 found in the lumen. In this protease-triggered cascade, trypsin-catalyzed release of HD6 unmasks latent biological activity by enabling peptide self-assembly to form the nanonets that can entrap bacterial invaders.

## Results

### Identification of HD6 isoforms in human intestinal tissue and luminal fluid

Human Paneth cells express and release two α-defensins, HD5 and HD6.^6,7,17,28^ Immunogold transmission electron microscopy (TEM) of human ileal tissue using antibodies that selectively react with either HD5 or HD6 demonstrated that HD5 and HD6 are co-packaged in Paneth cell secretory granules (Fig. 1b and S1a†). Because preliminary dot blots revealed that the HD6 antibody (generated to the 32-residue C-terminal peptide^17^) reacts with both recombinant proHD6 and mature HD6 (Fig. S1a†), the immunogold labeling did not elucidate which isoform(s) of HD6 was present in the Paneth cell granules, and prior work precludes prediction between these storage forms. For example, mouse Paneth cell α-defensins have an acidic propeptide (pI ≈ 4.5) and are stored as mature α-defensin peptides in the secretory granules.^25,26^ Similarly, human α-defensins 1–4 (also termed human neutrophil peptides 1–4, HNP1–4) have acidic propeptides (pI ≈ 5.5) and are stored in human neutrophil granules as the mature forms (29–30 amino acids).^20–23^ In contrast, HD5 has a cationic propeptide (pI ≈ 9.5) and is stored in Paneth cell granules as a propeptide.^24^ Therefore, uncertainty surrounded whether HD6, which has an acidic propeptide (pI ≈ 4.5), is stored in human Paneth cell granules as a mature peptide, like mouse Paneth cell α-defensins and human neutrophil α-defensins, or as a propeptide like HD5 despite its significantly different pI.

We therefore obtained human ileal tissue from surgical specimens and prepared protein extracts for analysis by Western blot, mass spectrometry, and Edman degradation. Western blot (AU-PAGE, acid urea–polyacrylamide gel electrophoresis) analysis of the protein extracts revealed a single band with HD6 immunoreactivity (Fig. 1c). MALDI-TOF mass spectrometry revealed a *m*/*z* value of 8962.1, which is in agreement with the calculated *m*/*z* value of 8960.2 for the oxidized form of proHD6 (Table S2†). Four rounds of Edman degradation afforded an N-terminal sequence of EPLQ that is in agreement with the N-terminus of the propeptide predicted from mRNA analysis (Fig. 1c). Data consistent with these findings were obtained from specimens of three individuals. These results established that HD6 is stored in Paneth cells as proHD6, an 81-residue propeptide, corresponding to residues 20–100 of the deduced preproHD6 sequence (Table S1, ESI†). We found no evidence for the presence of mature HD6 in the tissue samples examined in this study.

Next, to ascertain which isoform(s) of HD6 is present in the small intestinal lumen, we analyzed intestinal luminal aspirates obtained by endoscopy. Western blot (AU-PAGE) of the luminal fluid revealed a single band of HD6 immunoreactivity (Fig. 1d). We fractionated the luminal fluid by HPLC and screened the resulting fractions using HD6 immunoreactivity (dot blot) and MALDI-TOF mass spectrometry. We detected only one HD6 isoform characterized by a *m*/*z* value of 3709.9, which corresponds to the oxidized form of the 32-residue mature peptide (calculated *m*/*z* 3708.1 for residues 69–100 of the prepropeptide deduced from mRNA analysis) (Fig. 1d, S1 and Table S2†). This assignment was confirmed by seven rounds of Edman degradation, which afforded the N-terminal sequence AFTCHCR (Fig. 1d). Moreover, this HD6 isoform was the only one detected by MALDI-TOF mass spectrometry from luminal fluid specimens obtained from a total of six individuals.

A comparison of the N-terminal residues of the luminal HD6 peptide with the deduced cDNA sequence^7^ indicated that proteolytic processing of the propeptide occurred on the C-terminal end of Arg^68^ (Fig. 1a). In agreement with our hypothesis, this cleavage site is consistent with trypsin-catalyzed hydrolysis of the amide bond linking Arg^68^ and Ala^69^. Previous studies reported that trypsin is expressed by human Paneth cells,^24,27^ and demonstrated that trypsin processes proHD5 to release the 32-residue mature form.^24,27^ A definitive experiment to show co-packaging of this protease and either of these α-defensins in Paneth cell granules has not been reported, however. We extended our immunogold co-labeling studies of human ileal tissue to include trypsin, and confirmed that human enteric α-defensins and trypsin are co-packaged in the granules (Fig. 1b). In total, our analyses of HD6 in human ileal tissue and luminal fluid support a model whereby the peptide is stored in the secretory granules of Paneth cells as an 81-residue propeptide. Either during or after granule release into the lumen, proHD6 is cleaved by trypsin to generate HD6, the mature 32-residue peptide found in the intestinal lumen (Fig. S2†).

### Trypsin-catalyzed proteolysis of proHD6 provides mature HD6

To enable *in vitro* studies of proHD6, we obtained recombinant proHD6 following expression of His_6_-SUMO-proHD6 in *Escherichia coli*. This affinity tag allowed the first recombinant preparation and purification of native 81-residue proHD6. Ulp-1 digestion of His_6_-SUMO-proHD6 afforded proHD6 as a mixture of species resulting from peptide oxidation and disulfide bond formation. A sample of the oxidized form, the regioisomer exhibiting the three native α-defensin disulfide linkages (Cys^4^–Cys^31^, Cys^6^–Cys^20^, Cys^10^–Cys^30^), was obtained following chemical reduction of the peptide to yield the reduced form with six free thiols, oxidative folding, and purification (Table S2 and Fig. S3†). We conducted antimicrobial activity assays with proHD6 (Fig. S4†) and determined that proHD6 does not exhibit antibacterial activity against *Escherichia coli* ATCC 25922 or *Listeria monocytogenes* ATCC 19115, two strains employed in this work.

**Fig. 2:**
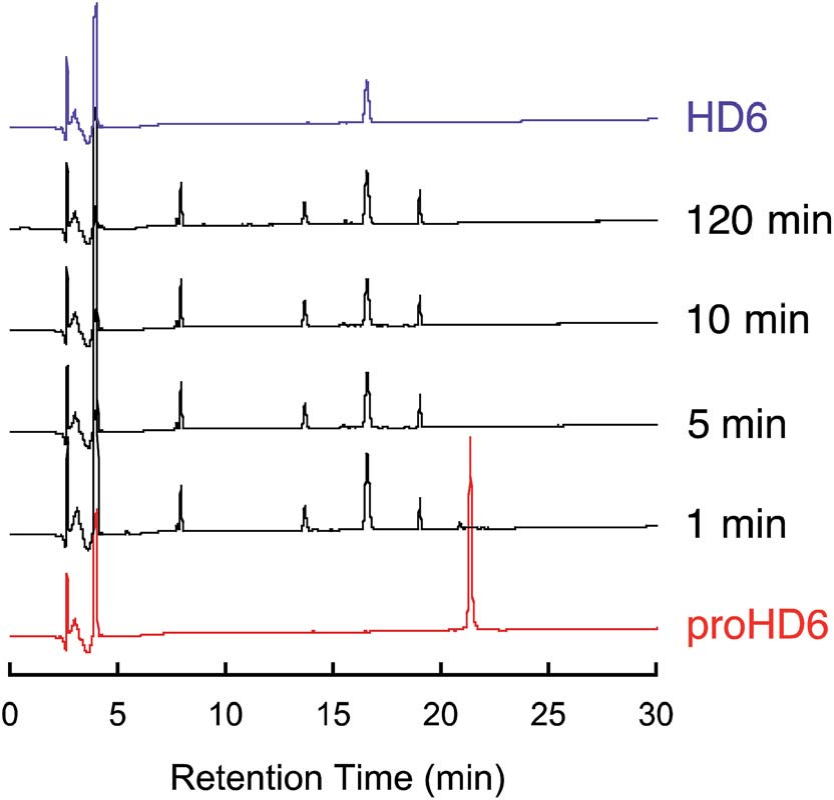
*In vitro* trypsin-catalyzed cleavage of proHD6. Analytical HPLC traces of trypsin-treated proHD6 (30 μM × 80 μL) at indicated time points. The trypsin concentration was 0.01 mg mL^–1^ (1 : 100 trypsin : proHD6 (w/w)). Absorbance at 220 nm was monitored with a reference wavelength of 500 nm. Method: 10–60% B over 30 min at 1 mL min^–1^. The HPLC trace of HD6 is shown in purple as a standard. Full analysis of the product peaks is given in Fig. S6.†

To test our hypothesis that trypsin-catalyzed hydrolysis of proHD6 affords mature HD6, we conducted *in vitro* proteolysis assays (Fig. 2 and S5†). Under the conditions of this experiment (1 : 100 trypsin : proHD6 (w/w) in 100 mM Tris–HCl, 20 mM CaCl_2_, pH 8.0), trypsin accepted proHD6 as a substrate, and cleavage of the propeptide was observed. Analytical HPLC and LC/MS of the product mixture revealed HD6 as well as a number of peptides corresponding to fragments of the propeptide domain (Fig. S6†). Moreover, these assays confirmed that the mature 32-residue HD6 is resistant to trypsin-catalyzed degradation (Fig. S5†). This observation is consistent with previous studies of other defensins, which demonstrated that the disulfide array confers protease resistance.^13,14^ These data, coupled with the presence of trypsin and HD6 in Paneth cells, implicate trypsin as the processing enzyme for HD6 maturation; however, we cannot exclude the possibility that other proteases contribute to proHD6 cleavage after secretion.

### Trypsin cleavage of proHD6 unmasks latent bacterial agglutination activity

**Fig. 3:**
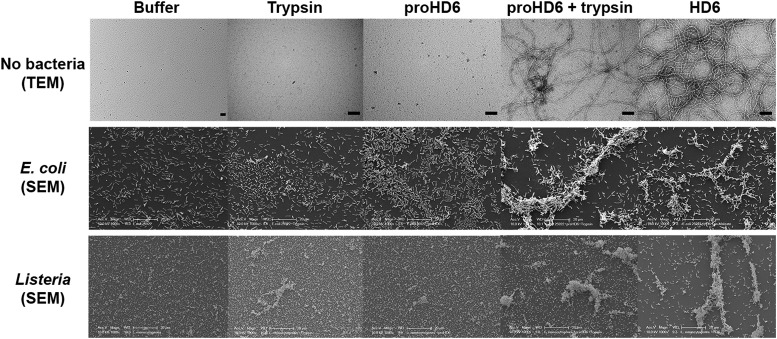
TEM analysis of HD6 self-assembly and SEM analysis of bacterial agglutination. Top row: transmission electron micrographs of 10 mM sodium phosphate pH 7.4 (control), 0.4 μM trypsin (control), 20 μM proHD6 in the absence and presence of 0.4 μM trypsin, and 20 μM HD6. All the samples were incubated at room temperature for 1 h. Scale bar = 100 nm. TEM images obtained using 10 mM Tris-maleate pH 6.4 are presented in Fig. S7.† Middle row: scanning electron micrographs of *E. coli* ATCC 25922 treated with 50 mM Tris-maleate pH 6.4 (control), 0.4 μM APMSF-inactivated trypsin (control), 3 μM proHD6, 3 μM trypsin-cleaved proHD6, or 3 μM HD6. Trypsin-cleaved proHD6 was prepared prior to incubation with the bacteria and the residual enzymatic activity was inhibited by APMSF (Experimental section). Following a 30 min incubation, the bacterial suspensions were centrifuged, fixed, and analyzed by SEM. Scale bar = 20 μm. Bottom row: SEM of *L. monocytogenes* ATCC19115 treated under the same conditions as in the middle row. Scale bar = 20 μm. Additional SEM images are shown in Fig. S9–S12.†

To probe whether proHD6 can self-assemble, we first imaged samples of proHD6 prepared in different buffers by transmission electron microscopy (TEM). The images of proHD6 were indistinguishable from the buffer-only controls, and we observed no evidence for the formation of higher-order oligomers (Fig. 3 and S7†). This property sharply contrasts with that of mature HD6, which forms extended fibrils that are >1 μm in length under the same experimental conditions (Fig. 3 and S7†). Moreover, when we added trypsin to proHD6 prior to TEM, we observed fibril-like features comparable to those observed for the mature HD6 peptide (Fig. 3 and S7†). Time-course experiments revealed that the fibril length increased with longer trypsin treatment (Fig. S8†). After a 5 min incubation with trypsin, relatively short fibrils were observed, whereas after 60 min, the fibrils closely resembled those of mature HD6. This experiment indicates that proHD6 cannot self-assemble into higher-order oligomers, but that trypsin-catalyzed hydrolysis of the propeptide affords this functional activity.

Next, we investigated the bacterial agglutination properties of proHD6 using scanning electron microscopy (SEM). Previous investigations demonstrated that mature HD6 agglutinates Gram-negative^15,17^ and Gram-positive bacteria.^15^ Treatment of *E. coli* ATCC 25922 or *E. coli* Nissle with proHD6 (3 μM) yielded no distinguishable agglutination. The bacteria appeared comparable to the buffer-only control and nanonets were not observed (Fig. 3, S9 and S10†). Treatment of proHD6 with trypsin prior to incubation with bacteria resulted in entangled and agglutinated *E. coli* in the SEM images, indistinguishable from the agglutination observed for *E. coli* treated with mature HD6 (Fig. 3, S9 and S10†). We found that treatment of *S*. Typhimurium, another Gram-negative bacterium, with mature HD6 resulted in entangled and agglutinated bacteria (Fig. S11†), consistent with previous studies.^17^ As observed for *E. coli*, proHD6 did not agglutinate *S*. Typhimurium unless it was treated with trypsin prior to incubation with the bacteria (Fig. S11†). Finally, we observed the same trypsin-dependent activity of proHD6 with the Gram-positive bacterium *L. monocytogenes* (Fig. 3 and S12†). Thus, the SEM investigations provide evidence that mature HD6 readily agglutinates both Gram-negative and Gram-positive bacteria, whereas proHD6 lacks this activity until it is processed by a protease.

**Fig. 4:**
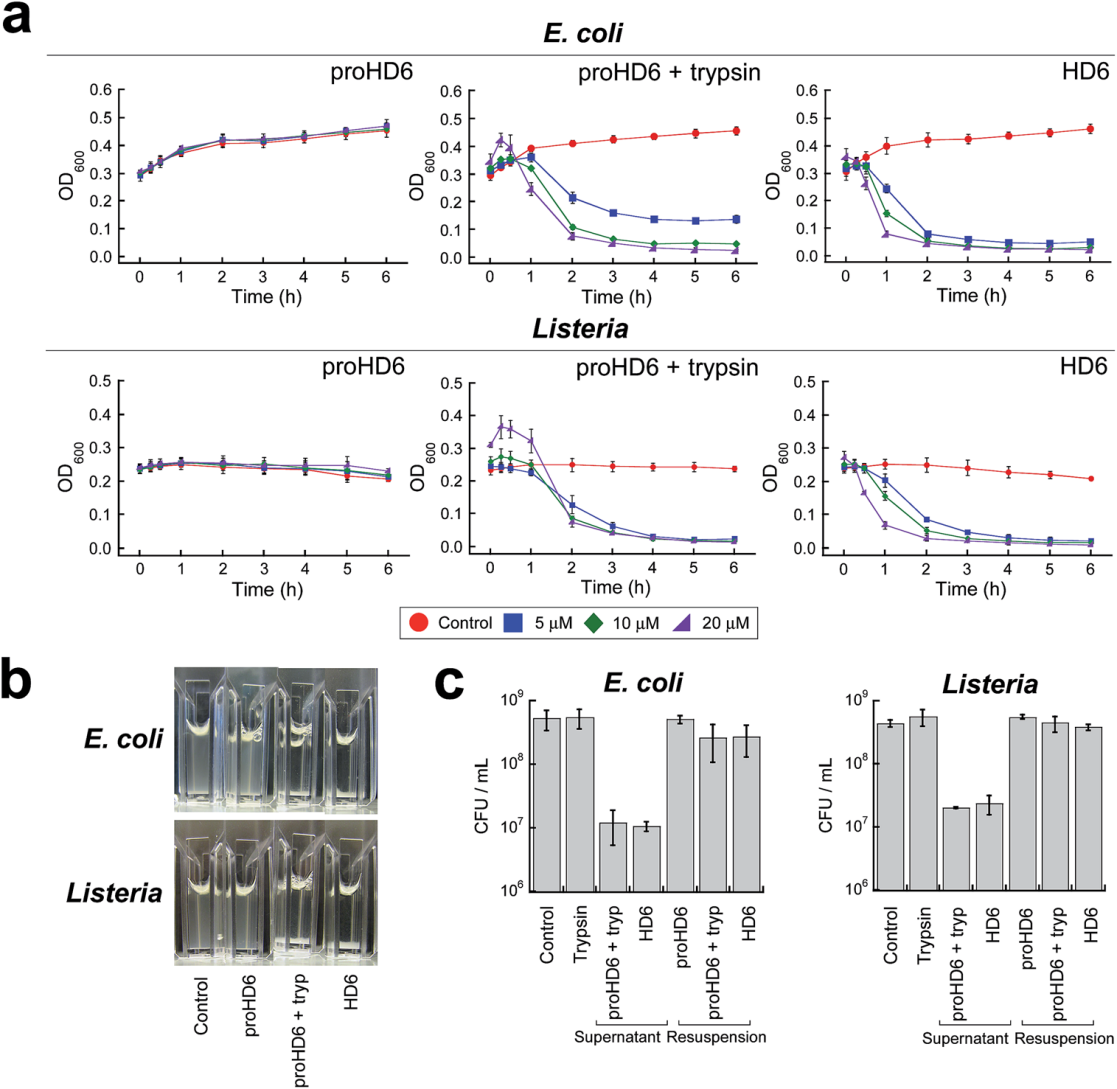
Bacterial agglutination assays for native and cleaved proHD6. (a) Agglutination of *E. coli* ATCC 25922 and *L. monocytogenes* ATCC 19115 in 50% MHB treated with proHD6 in the absence and presence of 0.4 μM trypsin or HD6 (control). (b) Representative images of cuvettes containing *E. coli* ATCC 25922 or *L. monocytogenes* ATCC 19115 after incubation with 20 μM peptides for 2 h at room temperature. (c) Plots of colony forming units (CFU mL^–1^) of *E. coli* ATCC 25922 and *L. monocytogenes* ATCC 19115 after treatment with 20 μM peptides for 6 h (mean ± SDM, *n* = 3). Supernatant is defined as the clarified medium solution in the top portion of the culture and resuspension is defined as the mixture after thoroughly agitating the heterogeneous solution.

We previously reported a simple cuvette-based *in vitro* agglutination assay that enables time-dependent monitoring of agglutination of viable bacteria by HD6.^15^ We employed this assay to further characterize the trypsin-dependent activity of proHD6 on cultured bacterial cells (Fig. 4a and b). When proHD6 was added to a suspension of either *E. coli* or *L. monocytogenes* (10^8^ CFU mL^–1^), the cultures remained homogeneous for the 6 h duration of the assay, even at the highest concentration of proHD6 evaluated (20 μM). In contrast, bacterial agglutination and sedimentation occurred when a combination of proHD6 and trypsin was added to the bacterial cultures (Fig. 4a and b), with kinetics comparable to those obtained for cultures treated with mature HD6. In these assays, we defined the “supernatant” as the culture solution in the top portion of the cuvette and the “re-suspension” as the mixture that results from agitating the entire culture after the 6 h incubation period.^15^ We observed an ≈1.5-fold log reduction in CFU mL^–1^ for the “supernatant” from cultures treated with combination of trypsin and proHD6 relative to the no-peptide control (Fig. 4c). Following “re-suspension”, the total CFU mL^–1^ in the cuvette was indistinguishable from that of either the untreated control culture or the bacterial culture treated with proHD6. These results indicated that the reduction of CFU mL^–1^ in the “supernatant” for the cultures treated with proHD6 and trypsin was a result of bacterial agglutination and sedimentation rather than bacterial cell death.

### Proteolysis of proHD6 protects human epithelial cells from *Listeria* invasion

**Fig. 5:**
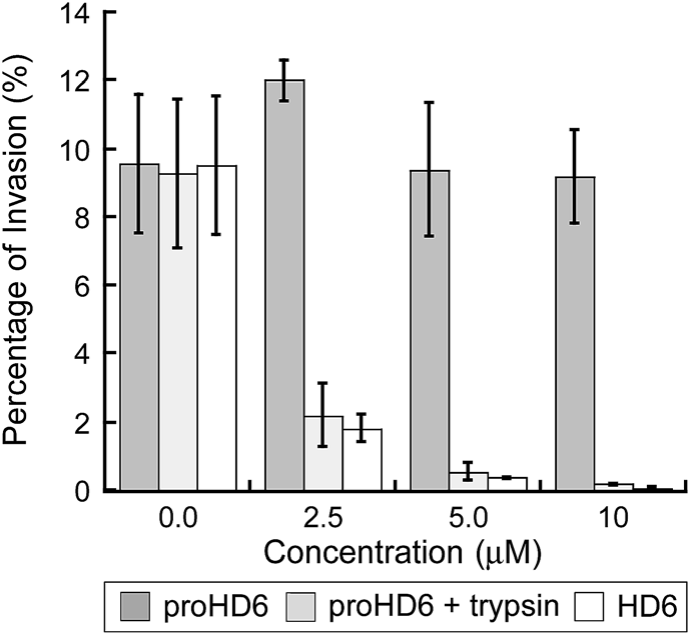
*Listeria* invasion assays for native and cleaved proHD6. Invasion of human T84 colon epithelial cells by *L. monocytogenes* ATCC 19115 pre-treated with proHD6 in the absence and presence of 0.4 μM trypsin or HD6 (control). The bacteria (2 × 10^6^ CFU mL^–1^) were incubated with the indicated peptides for 30 min prior to the infection of the T84 cells (mean ± SDM, *n* = 3).

Consistent with our recent study,^15^ mature HD6 (≥2.5 μM) blocks *L. monocytogenes* invasion into human intestinal epithelial cells (Fig. 5). In contrast to these findings with mature HD6, proHD6 (≤10 μM) did not impair the ability of *L. monocytogenes* to invade intestinal epithelial cells (Fig. 5). However, when proHD6 (≥2.5 μM) was treated with trypsin, the percentage of *L. monocytogenes* invasion decreased from ≈10% to <2%, similar to the decrease observed for equivalent concentrations of mature HD6 (Fig. 5). Taken together with the agglutination studies, these results indicate that trypsin-catalyzed hydrolysis of proHD6 triggers *L. monocytogenes* entrapment, which prevents this pathogen from invading mammalian cells. In control experiments, nuclear morphology assays indicated that neither proHD6 nor HD6 exert cytotoxic effects on the epithelial cells over the course of these assays (Fig. S13†).

### Structural determinants of proHD6 impair self-assembly

**Fig. 6:**
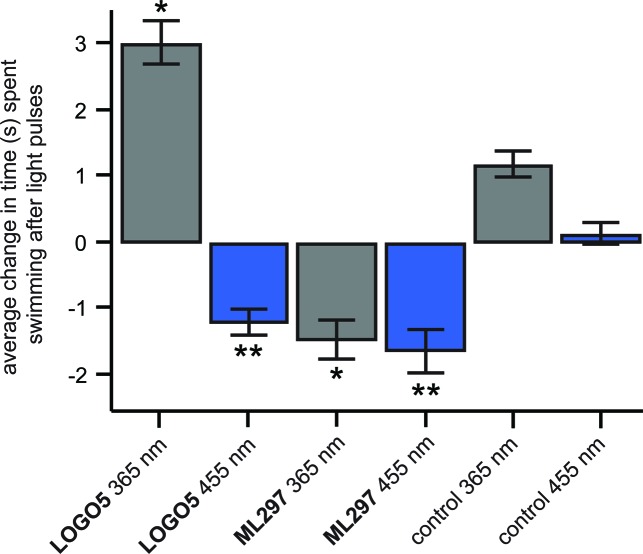
Biophysical characterization of proHD6. (a) CD spectra of 20 μM proHD6 (red) and 20 μM HD6 (purple) in 10 mM sodium phosphate buffer, pH 7.4. (b) Analytical ultracentrifugation of 140 μM proHD6 in 10 mM sodium phosphate buffer, pH 7.4. The blue dots are the –d*c*/d*t* data obtained from sedimentation velocity experiments (absorbance at 280 nm). The red line is the single Gaussian fit obtained using DCDT+. The summary of the fits is provided in Table S4.† (c) Sedimentation equilibrium profiles of 120 μM proHD6 in 10 mM sodium phosphate buffer, pH 7.4. Best fits (black lines) of raw UV absorbance at 280 nm at rotor speeds of 30 000 (red), 36 000 (blue), and 42 000 (green) rpm. The fits and calculated molecular weights are summarized in Table S5.†

The *in vitro* studies of proHD6 function obtained thus far suggest that this isoform is inactive as a result of suppressed self-assembly, leaving unanswered how the propeptide segment interferes with this process. Circular dichroism (CD) spectroscopy provides a fingerprint for mature HD6 oligomers because the CD signature of the HD6 self-assembly is characterized by a negative peak at 192 nm and positive peaks at 205 and 230 nm at pH 7.4 (Fig. 6a).^15^ Under the same conditions, the CD spectrum of proHD6 markedly differs with a negative peak at 197 nm, resembling the CD signatures of random-coils. Together with the initial TEM studies, these data provide biophysical evidence that the pro region interferes with the self-assembly process characteristic of mature HD6.

We employed analytical ultracentrifugation (AUC) to further evaluate the quaternary structure of proHD6. We determined the sedimentation coefficients of proHD6 prepared in different buffers (Table S4†). When we prepared proHD6 (≤140 μM) at pH 7.4 in 10 mM sodium phosphate buffer, a single peak at ≈ 1.8 S was obtained over the range *s*_20,w_ = 0.5–3.5 S in the Gaussian fits of the –d*c*/d*t* distributions obtained using DCDT+^29^ (Fig. 6b and S14a†). The Gaussian fits support the predominance of a single species of proHD6 over a concentration range of 30–140 μM at pH 7.4. Substitution of sodium phosphate with HEPES or Tris–HCl buffer at pH 7.4 had a negligible effect on the s value for proHD6 (Fig. S14b and Table S4†). In contrast to proHD6, attempts to evaluate mature HD6 by AUC were not successful because HD6 sedimented too rapidly even at the lowest speed that AUC can provide (3000 rpm) and coated the AUC cell.^15^ Nevertheless, AUC studies of mature HD6 variants that could not form fibrils observable by TEM yielded s values that confirmed their limited ability to oligomerize.^15^

Because this work includes the first isolation and biophysical evaluation of proHD6, there is no proHD6 crystal or solution structure available that can be used to calculate sedimentation coefficients. We therefore estimated the sedimentation coefficients of proHD6 (see Experimental section) under the assumption that it behaves as a smooth, compact, spherical peptide in water at 20 °C, which afforded values of 1.7 S (monomer) and 2.8 S (dimer). A caveat to this analysis is that the 49-residue N-terminal pro domain of proHD6 is likely dynamic, and it is unlikely that proHD6 behaves as a spherical peptide in aqueous solution.^30^ Nevertheless, comparison of the experimental and estimated s values suggests that proHD6 exists in monomer–dimer equilibrium with rapid association under these experimental conditions. We also conducted sedimentation equilibrium (SE) experiments to determine the molecular weight of proHD6 in solution (10 mM sodium phosphate, pH 7.4). Using SEDPHAT^31^ for a global analysis of the data, we obtained a molecular weight of 15 549 Da with a standard deviation of ±230 Da. At the 95% confidence level, the interval ranged from 15 059 Da to 15 946 Da using a Monte Carlo analysis of the fit (Fig. S15 and Table S5†). The molecular weights of a proHD6 monomer and dimer are 8961 and 17 922 Da, respectively. Thus, the experimental molecular weight of 15 549 Da indicates that the predominant proHD6 species under these conditions is a dimer and that monomer–dimer equilibrium occurs. Thus, these data are consistent with a model where proHD6 monomers can reversibly associate to form dimers, whereas mature HD6 readily self-assembles to form higher-order complexes. Proteolysis therefore serves as a biochemical switch to induce these pivotal biophysical characteristics.

## Discussion

In this work, we combined evaluation of HD6 localization and characterization in human intestinal specimens with *in vitro* functional studies and biophysical characterization to provide a model for the storage and maturation of this Paneth cell defensin. HD6 is an unusual defensin that lacks the typical broad-spectrum *in vitro* bactericidal activity of other defensins. It contributes to mucosal immunity by self-assembling into oligomeric structures, called nanonets, that entrap bacterial pathogens and block their invasion both *in vitro* and *in vivo*.^15,17^ This study illuminates a number of important facets pertaining to how HD6 is stored in the granules of Paneth cells and the mechanism of HD6 self-assembly in the intestinal lumen. First, our characterization of human ileal tissue demonstrates the co-packaging of HD5, HD6, and trypsin within the secretory granules of Paneth cells. Our analyses of ileal tissue protein extracts and luminal fluid enabled us to detect two distinct isoforms of HD6, the 81-residue propeptide that was only detected in the tissue samples and the 32-residue mature form that was only found in the luminal fluid. The N-terminal sequence of the mature HD6 peptide is consistent with cleavage of proHD6 by trypsin. On the basis of these observations and the primary sequence of proHD6, we proposed and tested a model whereby proHD6 is proteolytically cleaved to liberate mature HD6, which triggers its self-assembly in the gut. Our *in vitro* investigations show that trypsin-catalyzed hydrolysis of proHD6 generates the 32-residue defensin peptide and that this mature defensin is stable to further proteolysis. Moreover, our bacterial agglutination assays and biophysical characterization of proHD6 further inform this model by demonstrating that proHD6 cannot self-assemble into high-order oligomers or cause bacterial entrapment. Our work thus establishes that Paneth cells package HD6 in an inactive form and that proteolysis triggers its innate immune activity in the intestine. This process provides spatial and temporal control of peptide self-assembly and thereby unveils the protective properties of this molecule at the intestinal mucosa.

The observed co-packing of defensin propeptides and trypsin in human Paneth cell granules indicates that trypsin is inactive at this biological site. The mechanism underlying this lack of trypsin activity is currently unknown and a topic for future investigation. Spatial and temporal control of protease activity can be achieved by alterations in local pH or calcium ion concentration, the presence of protease inhibitors, and storage as a zymogen. Along these lines, human Paneth cells produce α_1_-antitrypsin,^24,32^ and this protein may inhibit trypsin packaged in the granules. We also reason that trypsin might be stored in the granules as a zymogen, trypsinogen, and activated by enterokinase or an enterokinase-like enzyme following release into the lumen. This model is based on the known processing of trypsinogen produced by pancreatic exocrine cells in the human intestine.

Whereas most α-defensins crystallize as dimers, the crystal structure of 32-residue HD6 revealed that a hydrophobic pocket occurs between four HD6 monomers.^12,15^ In each hydrophobic pocket, two monomers each contribute Phe^2^, Phe^29^ and Leu^32^, and the two other monomers each contribute Val^22^, Met^23^ and Ile^25^. The N- and C-termini of 32-residue HD6 are within close proximity, and residues at both the N- and C-terminal ends are constituents of this hydrophobic pocket. On the basis of the analytical ultracentrifugation studies presented here, the N-terminal pro-region prevents formation of this hydrophobic pocket and thereby self-assembly to higher-order oligomers. Elucidating whether the pro-region merely provides a steric block to self-assembly or whether specific amino acid side-chains in the pro-region are required for preventing HD6 oligomerization needs further biophysical investigations.

Our understanding of the roles of defensin propeptides and defensin maturation is limited, and the insights from the current work may also be considered in the context of reported observations about the proteolytic processing and maturation of other α-defensins.^24–26,33,34^ Although conversion from a propeptide to a mature form is common amongst α-defensins, the pro-regions of α-defensins exhibit variable primary sequences, and the maturation pathways and proteases involved in propeptide processing are oftentimes unknown. On the basis of characterized systems, some defensins are packaged as propeptides whereas others are stored in the mature forms, making it necessary to examine speciation on a case-by-case basis. A comparison of the human α-defensins illustrates this point. Previous studies^24,35^ and the current work demonstrate that the enteric α-defensins HD5 and HD6 are stored in Paneth cell granules as propeptides. In contrast, the human neutrophil peptides (HNP1–4) are stored in neutrophil granules as the mature, ≈30-residue peptides.^20–22^ The HD5 propeptide is highly cationic (pI ≈ 9.5) whereas the HD6 and HNP propeptides are anionic (pI ≈ 4.5 for HD6; pI ≈ 5.5 for HNPs). These comparisons indicate that the amino acid composition of the propeptide region does not provide a reliable predictor of whether a given peptide will be processed before or after packaging into granules, at least based on our current understanding. Moreover, prior studies of human ileal fluid detected several different isoforms of HD5 where the N-terminal proregion was truncated at different positions as a result of proteolytic processing at different sites, and all of these species displayed antibacterial activity.^24^ This observation contrasts with the current results for HD6, where homogeneous immunoreactivity was observed by AU-PAGE and only the 32-residue mature HD6 was detected in ileal fluid. Lastly, mice also harbor enteric α-defensins that are packaged in Paneth cell granules. Like human α-defensins, the murine α-defensins are translated as propeptides. In contrast to HD5 and HD6, the murine α-defensins are packaged in Paneth cell granules as the mature peptide and matrix metalloproteinase 7 has been implicated as the processing enzyme.^25,26^ Taken together, nature has deployed a variety of strategies for defensin maturation even within a particular class for reasons that are as-yet undetermined and warrant exploration in future work.

At mucosal surfaces throughout the body, numerous host peptides and proteins boost barrier effectiveness of the epithelia. By inhibiting bacterial invasion, HD6 contributes to barrier function of the innate immune system at the intestinal mucosa, augmenting the contributions of other key biomolecules at this surface such as mucus^36^ and lectins.^37^ The adaptive immune system also contributes to barrier function by using immunoglobulin A, which is abundant in the intestine and other mucosal surfaces, to block bacterial invasion.^38–40^ Thus, the combined invasion-inhibiting activities of the innate and adaptive immune systems fortify the integrity of the intestinal barrier to mediate intestinal homeostasis. Further investigations are required to elucidate additional mechanistic details pertaining to how HD6 performs its host-defense function. In particular, how HD6 function integrates with other host-defense molecules deployed at the intestinal mucosa requires investigation, and whether HD6 affects the population dynamics of human gut commensal organisms under normal or pathological conditions is another important avenue for future work.

In closing, propeptides are employed as inactive precursors to suppress the biological functions of various peptides and proteins, and proteases are enlisted to unleash the activity of the mature isoforms on demand.^24,41,42^ The N-terminal extension from mature HD6 is a fascinating variation on this general theme, which allows for suppression of peptide self-assembly and storage of HD6 in Paneth cells as an inactive form. The protease-triggered self-assembly of HD6 is reminiscent of neuropeptides that form higher-order oligomers following a protease cleavage event, including the prion protein and Aβ(1–42).^43–45^ In such cases, oligomer formation is associated with disease.^46,47^ Protease-triggered self-assembly of HD6, in contrast, affords a beneficial outcome to intestinal homeostasis by spatial and temporal control of its host-defense function entrapping bacterial invaders.

## Experimental section

### Materials and general methods

All solvents, reagents, and chemicals were purchased from commercial suppliers and used as received unless noted otherwise. The mature 32-residue forms of HD5 and HD6 were recombinantly overexpressed and purified as previously described.^14,15^ These procedures involve oxidative folding, which allows for isolation of the native Cys^I^–Cys^VI^, Cys^II^–Cys^IV^, Cys^III^–Cys^V^ regioisomers following purification. All functional and biophysical studies were performed with oxidatively folded peptides. Milli-Q water (18.2 mΩ cm) was passed through a 0.22 μm filter before it was used to prepare all buffers, aqueous solutions, and peptide/oligonucleotide stock solutions. Oligonucleotide primers were synthesized by Integrated DNA Technologies (Coralville, IA) and used as received (standard desalting protocol). A Biorad MyCycler thermocycler was employed for all polymerase chain reactions (PCR). Chemically-competent *E. coli* TOP10 and BL21(DE3) cells were prepared in-house *via* standard protocols. PfuTurbo DNA polymerase was purchased from Agilent Technologies. T4 DNA ligase and all restriction enzymes were purchased from New England Biolabs. DNA sequencing was performed by staff in the Biopolymers Facility at the Massachusetts Institute of Technology.

### Instrumentation

Unless noted otherwise, analytical and semi-preparative high-performance liquid chromatography (HPLC) were performed on an Agilent 1200 instrument equipped with a thermostatted autosampler set at 4 °C and thermostatted column compartment generally set at 20 °C, and a multi-wavelength detector set at 220 and 280 nm (500 nm reference wavelength with 100 nm bandwidth). Preparative HPLC was performed using an Agilent PrepStar 218 instrument outfitted with an Agilent ProStar 325 UV-visible dual-wavelength detector set at 220 and 280 nm. A Clipeus C18 column (5 μm particle size, 4.6 × 250 mm, Higgins Analytical, Inc.) set at a flow rate of 1 mL min^–1^ was employed for all analytical HPLC experiments. A ZORBAX C18 column (5 μm particle size, 9.4 × 250 mm, Agilent Technologies, Inc.) set at a flow rate of 5 mL min^–1^ was employed for all semi-preparative-scale HPLC purification. A Luna 100 Å C18 LC column (10 μm particle size, 21.2 × 250 mm, Phenomenex) set at a flow rate of 10 mL min^–1^ was utilized for all preparative-scale HPLC purification. HPLC-grade acetonitrile (MeCN) and HPLC-grade trifluoroacetic acid (TFA) were routinely purchased from EMD and Alfa Aesar, respectively. For all HPLC runs, solvent A was 0.1% TFA/H_2_O and solvent B was 0.1% TFA/MeCN.

High-resolution mass spectrometry was performed by using an Agilent LC-MS system comprised of an Agilent 1260 series LC system outfitted with an Agilent 6230 TOF system housing an Agilent Jetstream ESI source. For all LC-MS analyses, solvent A was 0.1% formic acid/H_2_O (LC-MS grade, Sigma-Aldrich) and solvent B was 0.1% formic acid/MeCN (LC-MS grade, Sigma-Aldrich). A Poroshell 120 EC-C18 column (2.7 μm particle size, 2.1 × 100 mm, Agilent Technologies, Inc.) set at a flow rate of 0.4 mL min^–1^ was employed for all LC-MS analyses of HD6. The samples were analyzed by using a gradient of 5–95% B over 5 min. A Poroshell 300SB C18 column (5 μm particle size, 2.1 × 75 mm, Agilent Technologies, Inc.) set at a flow rate of 0.2 mL min^–1^ was employed for all LC-MS analyses of proHD6. The samples were analyzed by using a gradient of 5–65% B over 30 min. The MS profiles were analyzed and deconvoluted by using Agilent Technologies Quantitative Analysis 2009 software version B.03.02.

A Beckman Coulter DU 800 UV-visible spectrophotometer was employed for all routine optical absorption measurements and aggregation assays. Extinction coefficients (280 nm) were calculated by using ExPASy ProtParam. The calculated extinction coefficients of native HD6, proHD6, and His_6_-SUMO-proHD6 are 4845, 6335, and 7825 M^–1^ cm^–1^, respectively. Peptide stock solutions were routinely prepared in Milli-Q water and concentrations were quantified by using the calculated extinction coefficients. Solution and buffer pH values were verified by using a Mettler Toledo S20 SevenEasy pH meter or a HANNA Instruments HI 9124 pH meter equipped with a microelectrode. An Aviv Model 202 circular dichroism spectrometer operated at room temperature was utilized to collect CD spectra.

### Human specimen samples and ethics approval

The protocols for obtaining tissue specimens and luminal aspirates were reviewed and approved by the Institutional Review Board at the Cleveland Clinic Foundation (#06-673) and informed consent was obtained from all patients. All specimens were coded so that patient identifiers were removed and then handled as previously described.^17,24,48^ Nondiseased specimens of human distal small intestinal tissue were obtained from redundant surgically-resected tissue as described.^24^ Small intestinal lumen fluid aspirates were obtained from individuals undergoing colonoscopy for either routine colon polyp screening or for assessment of inflammatory bowel disease. Approximately 5–15 mL of ileal fluid was aspirated, immediately placed on dry ice, frozen, and stored at –80 °C prior to use.

### Immunogold labeling and transmission electron microscopy

Human ileal tissue specimens were fixed in 4% paraformaldehyde (EM Science, Hatfield, PA) overnight at room temperature. The tissue was then dehydrated in graded ethanol and thin-sectioned. Grids were incubated with rabbit polyclonal HD6 antiserum (1 : 1000),^17^ mouse monoclonal anti-HD5 IgG (HyCult, Plymouth Meeting, PA), or rabbit polyclonal trypsin antiserum (Abcam, Cambridge, MA) for 1 h at room temperature in a humidified chamber, washed with PBS, and incubated with 1 : 50 dilution of 5 or 15 nm gold-labeled goat-anti-rabbit or goat-anti-mouse antibody (EM Science, Hatfield, PA) for 30 min. Grids were washed with double-distilled water and visualized on a Philips CM120 Biotwin Lens (F.E.I. Company, Hillsboro, OR) with Gatan BioScan, model 792 (Pleasanton, CA).

### HD6 peptide isolation from intestinal tissue

Protein extraction and fractionation from small intestinal tissue followed a published protocol,^24^ with some modifications. Briefly, approximately 1 g of human ileal tissue was homogenized with a Brinkmann Polytron homogenizer in ice-cold 20% acetic acid (1 : 10 w/v) that contained 1 : 100 v/v Protease Inhibitor Cocktail III. The extract was stirred overnight at 4 °C, and then clarified by ultracentrifugation (110 000*g* × 30 min, 4 °C). Ammonium sulfate was added (final concentration of 25% w/v), and the mixture was stirred at room temperature for 1 h, and then clarified by ultracentrifugation (110 000*g* × 30 min, 4 °C). The supernatant was then dialyzed against 5% v/v acetic acid overnight at 4 °C using Spectro/Por dialysis membrane (1 kD MWCO, Spectrum Laboratories, Rancho Dominguez, CA). The resulting solution was passed through a strong cation exchange cartridge (Bio-Scale Mini Macro-Prep High Q, BioRad, Hercules, CA), washed with 5% v/v acetic acid, and then eluted with 1 M NaCl. The eluate was further purified by RP-HPLC using a Waters 650E HPLC instrument with a variable wavelength detector (monitored at 214 nm and 280 nm) and a C18 column (Vydec) with a gradient of 5–80% acetonitrile gradient in 0.1% TFA. Fractions were collected at a flow rate of 1 mL min^–1^ and analyzed as described for the lumen aspirate specimens.

### HD6 peptide isolation from intestinal lumen fluid

The frozen ileal fluid aspirate was thawed on ice, and a protease inhibitor cocktail was added at a ratio of 1 : 100 v/v (Cocktail III, Calbiochem, La Jolla, CA). The resulting sample was immediately acidified with acetic acid (20% v/v final). Clumps and particulates were dispersed with a Brinkmann Polytron homogenizer. The sample was clarified by centrifugation at 29 000*g* (2 × 30 min, 4 °C). The supernatant was diluted with water (1 : 1 v/v), flash frozen, and then lyophilized to dryness. The lyophilized product was dissolved in 1.5 mL of 5% v/v acetic acid, and filtered through a Millex 0.22 μm polyether sulfone filter (EMD-Millipore) to remove residual particulates. A portion of the sample was then fractionated by reverse phase HPLC (RP-HPLC) using a Waters 650E instrument outfitted with a C18 column (10 μm particle size, 4.6 × 250 mm, Vydac/Grace, Columbia MD) using a gradient of 5–62% B over 60 min at a flow rate of 1 mL min^–1^. Fractions were collected at 1 min intervals and absorbance at 220 and 280 nm was monitored. Aliquots of each fraction were analyzed by MALDI-TOF mass spectrometry (2 μL aliquot) and for immunoreactivity (20 μL aliquot). Fractions positive for HD6 were then analyzed by Edman degradation as described below.

### MALDI mass spectrometry

A 4700 MALDI TOF/TOF (Applied Biosystems, Foster City, CA) equipped with a pulsed Nd:YAG laser (337 nm) and a delayed extraction ion source was employed to screen the HPLC fractions for HD6 isoforms. An aliquot of each fraction (2 μL) was mixed with an equal volume of a saturated matrix solution (α-cyano-4-hydroxycinnamic acid in 0.1% TFA/50% MeCN). A 1 μL aliquot of this mixture was applied to the target plate. The mass spectra were acquired in linear mode and typically ≈1000 laser shots were used per sample.

### Dot blot immunoreactivity

A dot blot method was used to screen HPLC sample fractions for HD6 immunoreactivity. A 20 μL aliquot of each fraction was spotted directly onto an Immobilon PSQ PVDF membrane (Millipore, Billerica, MA). The protein on the membrane was then fixed with 0.01% w/v glutaraldehyde (Sigma, St. Louis, MO) for 2.5 min, washed in Tris-buffered saline for 1 min, and the membrane surface was blocked with 5% w/v non-fat dry milk for 1 h. The membrane blots were probed with HD6 rabbit polyclonal antibody^17^ (1 : 7000) using a horseradish peroxidase (HRP)-conjugated goat-anti-rabbit secondary antibody (KPL, Gaithersburg Maryland). Signal was detected using Immobilon Western Chemiluminescent HRP substrate (Millipore, Billerica, MA). The chemiluminescent signal was detected with a Biospectrum AC Imaging System (UVP, Upland, CA).

### Edman degradation

Sample fractions with the positive HD6 immunoreactivity and mass consistent with (pro)HD6 were analyzed by Edman degradation to determine N-terminal amino acid sequence. The analysis was performed with a Procise 494 microsequencer (Applied Biosystems) by staff at the UC Davis Molecular Structure Facility.

### Western blot analysis

Acid urea–polyacrylamide gel electrophoresis (AU-PAGE) Western blot analysis was performed on small intestinal tissue and luminal fluid as previously described.^19,24^ Briefly, small intestinal ileal specimens (tissue and luminal aspirate fluid) were homogenized with a Brinkmann Polytron homogenizer in ice-cold 20% acetic acid (1 : 10 w/v) that contained 1 : 100 v/v Protease Inhibitor Cocktail III. The resulting suspension was stirred overnight at 4 °C and clarified the following day by ultracentrifugation (110 000*g* × 30 min, 4 °C). Aliquots of these specimens were diluted with 0.5 volume of loading buffer (9 M urea, 5% v/v acetic acid, 0.1 mg mL^–1^ methyl green (Sigma)) and then resolved on polyacrylamide gels (12.5% acrylamide/2% bis-acrylamide (Roche, Indianapolis, IN), 8 M deionized urea (Sigma), and 5% v/v acetic acid). Samples were run toward the cathode (reverse typical polarity) at 130 volts in 5% v/v acetic acid running buffer until the methyl green indicator dye reached the bottom of the gel (typically ≈1.5 h). Proteins were then transferred from the gels to Immobilon PSQ PVDF membranes in 5% v/v acetic acid using a semi-dry transfer apparatus (Fisher Scientific, Pittsburgh, PA) at 1.5 mA cm^–2^ toward the cathode for 20 min. Each membrane was then fixed, washed, blocked, and probed as described for the dot blot analysis.

### Subcloning, overexpression, and purification of His_6_-SUMO-proHD6

The pET SUMO-*proHD6* plasmid was prepared by using the Champion pET SUMO protein expression system (Invitrogen). The proHD6 nucleotide sequence (246 bp) was PCR amplified using pET28b-*proHD6* (ref. 15) as a template and the forward and reverse primers 5′-GAGCCGCTGCAAGCAGAG-3′ and 5′-*TTA*CAGACAACAAAAGCGATG-3′, respectively (stop codon, italic). The PCR products were analyzed by 1% (w/v) agarose gel and purified by using a QIAquick PCR purification kit (Qiagen). The PCR-amplified *proHD6* gene was subsequently ligated into pET SUMO using T4 DNA ligase (9 ng of PCR product, 50 ng of linear pET SUMO, 1 μL of T4 DNA ligase; 16 h, 16 °C). A 1 μL aliquot of each ligation reaction was transformed into chemically-competent *E. coli* Mach1™-T1^R^ cells (Invitrogen). The plasmids were isolated by using QIAprep spin miniprep kit (Qiagen) and the plasmid identities were verified by DNA sequencing.

The overexpression and purification of His_6_-SUMO-proHD6 were modified from the procedure for obtaining His_6_-proHD6.^15^ pET SUMO-*proHD6* was transformed into chemically-competent *E. coli* BL21(DE3) cells. Overnight cultures were prepared by inoculating LB medium containing kanamycin (50 μg mL^–1^) with single colonies. These cultures were grown to saturation (37 °C, 150 rpm, 16–18 h) and used to prepare freezer stocks. The freezer stocks, containing a 1 : 1 ratio of the overnight culture and sterile-filtered 50% glycerol in Milli-Q water, were stored at –80 °C. For a given His_6_-SUMO-proHD6 overexpression, 50 mL of LB medium containing 50 μg mL^–1^ kanamycin in a 250 mL baffled flask was inoculated from the freezer stock and grown to saturation (37 °C, 150 rpm, 16–18 h). The resulting culture was diluted 1 : 100 into 2 L of fresh LB medium containing 50 μg mL^–1^ of kanamycin in a 4 L baffled flask and incubated at 37 °C, 150 rpm until OD_600_ of ≈0.6 was achieved. Subsequently, a 400 μL aliquot of 0.5 M isopropyl β-d-1-thiogalactopyranoside (IPTG) was added to the 2 L culture and the culture was incubated for an additional 4–5 h until OD_600_ reached 1.2–1.5. The cells were centrifuged (3000 rpm × 15 min, 4 °C) and the cell pellets were collected. Overexpression of His_6_-SUMO-proHD6 was usually performed on a 12 L scale and the cell pellets from 6 L of culture were combined in pre-weighed 50 mL polypropylene centrifuge tubes (≈2 g L^–1^ wet cell weight), flash frozen in liquid N_2_, and stored at –80 °C for a period of 1–2 months.

For purification of His_6_-SUMO-proHD6, each 6 L cell pellet was thawed on ice and resuspended in 40 mL of cold lysis buffer (6 M GuHCl, 100 mM Tris–HCl, pH 8.0). A 1 mL aliquot of phenylmethyl sulfonyl fluoride (PMSF, 100 mM in EtOH) was added to the resuspension and the cells were transferred to a pre-chilled stainless steel beaker and lysed on ice by two rounds of sonication (10% amplitude with pulse on for 1 s and pulse off for 4 s for 1 min, on ice, Branson sonicator). A second 1 mL aliquot of PMSF (100 mM) was added to the cell lysate followed by centrifugation (13 000 rpm × 30 min, 4 °C). The resulting supernatant was incubated with pre-washed Ni-NTA resin (Qiagen, from 9 mL of Ni-NTA slurry for a cell pellet from 6 L of culture that was pre-washed 3 × 30 mL with Milli-Q water) with gentle shaking for 1.5 h at 4 °C. The resulting mixture was then loaded onto a fritted glass column and the resin was washed with 40 mL of cold wash buffer (20 mM Tris–HCl, 300 mM NaCl, 6 M GuHCl, pH 8.0). The His_6_-SUMO-proHD6 fusion protein was eluted with 30 mL of cold elution buffer (10 mM Tris–HCl, 300 mM NaH_2_PO_4_, 200 mM NaCl, 1 M imidazole, 6 M GuHCl, pH 6.5). The eluent was diluted with 30 mL of Milli-Q water, transferred into a dialysis bag (3500 MWCO), and dialyzed (2 × 12 h) against refolding buffer (20 mM Tris–HCl, 150 mM NaCl, 10% v/v glycerol, 1% w/v CHAPS, pH 8.0). The solution of His_6_-SUMO-proHD6 was concentrated to 2 mg mL^–1^, transferred to 50 mL polypropylene centrifuge tubes, flash frozen in liquid N_2_ and stored at –80 °C. The average yield was 20 mg L^–1^ culture. The purity of His_6_-SUMO-proHD6 was routinely evaluated by SDS-PAGE (15% Tris–HCl gel). A representative gel is shown in Fig. S3a.†

### Preparation and purification of native proHD6_red_

To a solution of His_6_-SUMO-proHD6, tris-(2-carboxylethyl)phosphine (TCEP, 100 mM stock solution in Milli-Q water) was added to achieve a final concentration of 2 mM and the solution pH was adjusted to pH 8.0 by drop-wise addition of 4 M NaOH. An 1 mg mL^–1^ stock solution of Ulp1 in 20 mM Tris–HCl, 150 mM NaCl, pH 8.0 was added to the solution containing His_6_-SUMO-proHD6 to afford a His_6_-SUMO-proHD6 : Ulp1 ratio of 100 : 1 (w/w). The reaction was incubated at room temperature for 2 h and quenched by addition of 6% aqueous TFA (10% v/v). The quenched reaction was immediately vortexed and incubated on ice for 10 min, and a precipitate formed. The mixture was centrifuged (3750 rpm × 15 min, 4 °C), and the supernatant was decanted and saved. The precipitate was resuspended in 20 mL of 6 M GuHCl and passed through a 0.22 μm filter. Analytical HPLC and LC-MS revealed that the majority of the precipitate was reduced 81-residue proHD6 (proHD6_red_, Table S2†). The supernatant portion, which was saved from the cleavage reaction, was dialyzed (3500 MWCO) against Milli-Q water (2 × 12 h), lyophilized, and resuspended in 75 mM HEPES, pH 8.0 containing 6 M GuHCl and 2 mM TCEP. After 15 min incubation at room temperature, the solution was acidified with 6% aqueous TFA (10% v/v). Analytical HPLC and LC-MS revealed that proHD6 in the supernatant portion was completely reduced. Subsequently, proHD6_red_ from both supernatant and precipitate portions was purified by preparative HPLC using a solvent gradient of 33–38% B over 16 min at 10 mL min^–1^. The desired product eluted at 14.2 min and was lyophilized to afford a white powder (≈0.5 mg L^–1^ of culture, Table S2†).

### Oxidative folding

Reduced proHD6 was oxidatively folded to afford a single regioisomer with the native S–S bonds by modifying a literature procedure.^15,49^ A 6 mg portion of proHD6_red_ was dissolved in 1 mL of 8 M GuHCl containing 3 mM glutathione and 0.3 mM glutathione disulfide. Then, 3 mL of 250 mM NaHCO_3_ was added to the solution to raise the pH to ≈8.3 and afford a final peptide concentration of 1.5 mg mL^–1^. The mixture was incubated at room temperature for 16 h. The resulting solution was analyzed by HPLC and LC-MS to confirm that proHD6_red_ was completely converted to a single oxidized species. The solution was centrifuged (3750 rpm × 10 min, 4 °C), passed through a 0.22 μm filter, and purified by preparative HPLC using a solvent gradient of 32–37% B over 16 min at 10 mL min^–1^. The single regioisomer obtained by oxidative folding eluted at 13.1 min and was lyophilized, which afforded pure oxidized proHD6 as a white powder (≈0.2 mg L^–1^ of culture). Analytical HPLC indicated that proHD6 was obtained in high purity (Fig. S3b†). Thiol quantification confirmed that proHD6 had no free Cys residues and LC-MS confirmed its identity (Table S2†).

### Antimicrobial activity assays

The assays were performed according to a literature procedure.^15^ Briefly, a 90 μL aliquot of the diluted bacterial culture was added to each well and then to each well was added 10 μL of a 10× concentrated aqueous peptide solution (500 μM) or sterile Milli-Q water as a no-peptide control. The plate was incubated for 1 h (37 °C, 150 rpm). The serial dilution for CFU counting was conducted as previously reported.^15^ These assays were performed with at least two independently prepared and purified samples of each peptide and in three independent trials. The resulting averages and standard deviations are reported.

### Trypsin-catalyzed cleavage of proHD6

A solution of proHD6 (100 μM, 180 μL) was prepared in 100 mM Tris–HCl, 20 mM CaCl_2_, pH 8.0. A 1 mg mL^–1^ stock solution of TPCK-treated trypsin (Worthington) in Milli-Q water was added to the solutions to achieve a final concentration of 0.01 mg mL^–1^. The mixture was incubated at room temperature with gentle shaking. A 24 μL aliquot was taken at varying time points, diluted with 56 μL of Milli-Q water, and quenched with 8 μL of 6% aqueous TFA. The resulting samples were vortexed, centrifuged (13 000 rpm × 10 min, 4 °C), and analyzed by HPLC (10–60% B over 30 min at 1 mL min^–1^).

### Negative-staining transmission electron microscopy

The samples for TEM were prepared by following a literature protocol.^15^ For each sample, a 5 μL aliquot of peptide solution (20 μM in 10 mM sodium phosphate pH 7.4 or 10 mM Tris-maleate pH 6.4) was placed onto the carbon-coated surface of a copper grid (400 square mesh, Electron Microscopy Sciences). After 1 min, the grid was washed with a 5 μL aliquot of Milli-Q water, stained with a 5 μL aliquot of 2% uranyl acetate (UA, Electron Microscopy Sciences) in Milli-Q water three times, and air-dried for at least 15 min before imaging. A FEI Technai Spirit Transmission Electron Microscope was employed for all TEM imaging (W.M. Keck Microscopy Facility, Whitehead Institute, Cambridge, MA). TEM images were obtained with at least two independently prepared peptides and representative images are presented.

### Scanning electron microscopy

Bacteria were grown aerobically at 37 °C overnight in LB media. The next morning the strains were subcultured and grown to mid-logarithmic phase in LB media. An aliquot of the bacteria (0.5–2 × 10^7^ CFU mL^–1^) was removed, sedimented by centrifugation (7000*g* × 2 min, 4 °C), washed twice with 50 mM Tris-maleate buffer, pH 6.4, and then resuspended in 0.5 mL of the 50 mM Tris-maleate buffer. The bacterial suspension was incubated at room temperature for 30 min with buffer control, 0.4 μM APMSF-inactivated trypsin, 3 μM proHD6, 3 μM trypsin-cleaved proHD6, or 3 μM HD6. Trypsin-cleaved proHD6 was prepared by incubating 30 μM proHD6 with 4 μM trypsin (Affimetrix, Santa Clara, CA) for 1 h at room temperature in 50 mM Tris-maleate buffer, pH 6.4. At the end of incubation, the trypsin was inactivated by addition of APMSF (*p*-amidinophenylmethylsulfonylfluoride HCl, Millipore, 25 μg mL^–1^), and the mixture was added to the bacterial suspension. The APMSF-inactivated trypsin was similarly prepared, except that proHD6 was omitted from the preincubation. After a 30 min incubation, the treated bacterial suspensions were sedimented by centrifugation (7000*g* × 5 min, 4 °C), and the buffer supernatant was carefully removed. The bacterial pellet was then resuspended in 100 μL of Karnosky fixative (2% paraformaldehyde, 2.5% glutaraldehyde in 0.06 M Sorensen's phosphate buffer (0.2 M sodium phosphate, pH 7.2)). Scanning electron microscopy was performed as previously described.^17^

### Bacterial agglutination assays

Stock solutions (50 μL) of HD6 and proHD6 (0, 50, 100, and 200 μM; 10× concentrations) were prepared in sterile Milli-Q water. If trypsin was required, aliquots of a 1 mg mL^–1^ stock solution of TPCK-treated trypsin (Worthington) prepared in sterile Milli-Q water were added to solutions of proHD6 to afford a final trypsin concentration of 0.01 mg mL^–1^. The assays were then conducted following the reported procedure.^15^ All assays were conducted with at least two independently prepared and purified samples of each peptide and in three independent trials. The resulting averages with standard deviations are reported.

### *Listeria* invasion assays

Concentrated (20×, 10 μL) solutions of HD6 and proHD6 were prepared in sterile Milli-Q water (0, 50, 100, and 200 μM). For conditions that required trypsin, a 1 mg mL^–1^ stock solution of TPCK-treated trypsin (Worthington) in sterile Milli-Q water was added to the 20× proHD6 solutions to yield a final trypsin concentration of 0.01 mg mL^–1^. A 190 μL aliquot of the diluted bacterial culture was added to each 10× peptide solution and the resulting mixtures were incubated at room temperature for 30 min. The invasion assays were then performed by following a literature protocol.^15,50^ All invasion assays were conducted with at least two independently prepared and purified samples of each peptide and in three independent trials and the resulting averages with standard deviations are reported.

### Nuclear morphology assays

Nuclear morphology assays were conducted to monitor T84 cell viability qualitatively following 1.5 h incubation with HD6 or proHD6 following a literature protocol with modification.^51^ T84 cells were passed and 500 μL of cells at the density of 2 × 10^5^ cells per mL was added to each well of a 24-well plate, which contained 12 mm untreated glass coverslips 12–16 h before the assays. The 20 μM peptide solutions in 1 : 1 DMEM/F12 (200 μL) were added to the cells and the cells were incubated for 1.5 h at 37 °C, 5% CO_2_. Then, the medium was removed and the cells were washed (1 × 500 μL) with PBS and fixed for 5 min with 500 μL of PBS containing 4% paraformaldehyde and 4% sucrose. The cells were subsequently washed with PBS (2 × 500 μL) and bathed in 500 μL of PBS containing 800 nM Hoechst 33258 (Sigma Aldrich) for 5 min. The Hoechst solution was then removed. The cells were washed with 500 μL of PBS, bathed in PBS, and mounted onto glass slides using the Vectashield antifading reagent (Vector Labs). The samples were examined using Zeiss LSM 710 NLO laser scanning confocal microscope (W.M. Keck Microscopy Facility, Whitehead Institute, Cambridge, MA) and 60–70 cells were scored for each sample. The images were processed using ImageJ.

### Sedimentation velocity experiments

The experiments were set up as previously reported.^15^ In one set of experiments, 10× concentrated proHD6 solutions in Milli-Q water (≈40 μL) were transferred to microcentrifuge tubes and lyophilized to dryness. A 400 μL aliquot of 10 mM sodium phosphate buffer adjusted to pH 7.4 (pre-filtered, 0.22 μm filter) was added to each microcentrifuge tube to achieve the desired concentrations (30, 50, 100, and 140 μM), and transferred to AUC sample cells. The pH of each solution was measured to confirm that it remained unchanged. The samples were centrifuged at 42 000 rpm and 20 °C until sedimentation was complete. The absorption wavelength for optical detection was 280 nm. All SV experiments were conducted with at least two independently prepared and purified samples of each peptide and in at least two independent trials.

Additional SV experiments were conducted to evaluate the effect of buffer components on the sedimentation of proHD6. In all cases, the proHD6 samples (400 μL) were prepared as described above except that proHD6 was dissolved in 10 mM Tris–HCl or HEPES pH 7.4 to obtain a final peptide concentration of 50 μM.

The details of data analysis are reported elsewhere.^52^ SEDNTERP^53^ was employed to calculate the buffer viscosity (*η*), buffer density (*ρ*), and the partial specific volume (value of proHD6 at 20 °C). The theoretical sedimentation coefficients of the proHD6 monomer and dimer were calculated by employing eqn (1)1
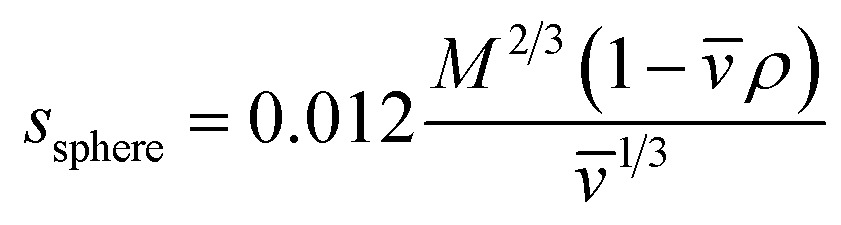
where *s*_sphere_ is the sedimentation coefficient for an ideal sphere in S units, *M* is the molar mass of the molecule of interest in Daltons, is in milliliters per gram, and *ρ* is in grams per milliliter. These calculations assume that proHD6 behaves as a smooth, compact, and spherical peptide in water at 20 °C.^30^

The experimental sedimentation coefficients were calculated by fitting the time derivative of the sedimentation velocity (–d*c*/d*t*) data using DCDT+.^29,54^ The –d*c*/d*t* distribution was generated from 22 to 28 scans with a peak broadening limit of 40 kDa by using DCDT+. The results are reported in Table S4.†

### Sedimentation equilibrium experiments

Sedimentation equilibrium (SE) experiments were performed to determine the molecular weight of proHD6. The proHD6 samples were prepared as described in the SV experiments with modification. In a typical set of experiments, proHD6 solutions in Milli-Q water (≈100 μL) prepared in microcentrifuge tubes were lyophilized to dryness. An aliquot (110 μL, pre-filtered, 0.22 μm filter) of 10 mM sodium phosphate buffer adjusted to pH 7.4 was added to each tube to afford solutions with varying concentrations of proHD6 (30, 60, and 120 μM) and transferred to AUC sample cells. The pH of each solution was measured to confirm that each solution remained at pH 7.4. Based on the sedimentation coefficients obtained from the SV experiments, equilibrium profiles were obtained at rotor speeds of 30 000, 36 000, and 42 000 rpm. Once equilibrium was established, two scans with five replicates were recorded. The absorption wavelength for optical detection was 280 nm, and the instrument was maintained at 20 °C.

The details of the experimental setup and data analysis are reported elsewhere.^52^ SEDNTERP^53^ was employed to calculate the buffer viscosity (*η*), buffer density (*ρ*), and the partial specific volume (value of proHD6 at 20 °C) as described in the SV section. The molecular weight of proHD6 was determined by global fitting of the multispeed equilibrium data across all loading concentrations at pH 7.4 using SEDPHAT.^31^ The Species Analysis model with mass conservation was employed for data analysis. The bottom of the sample sector was assigned as a floating parameter. To further evaluate whether each least-squares curve-fitting procedure converged to a global minimum, the alternate methods of Simplex, Marquardt–Levenberg, and simulated annealing were employed to assess any change in the global reduced chi-squared value.

### Circular dichroism spectroscopy

Peptide solutions (20 μM, 300 μL) were prepared in 10 mM sodium phosphate buffer, pH 7.4 and transferred to a 1 mm path-length quartz CD cell (Hellma) for all measurements. The CD spectra were collected from 260–190 nm at 1 nm intervals (5 s averaging time, three independent scans per wavelength). The data obtained from the three scans were averaged and the resulting averaged spectra are reported.

### Thiol quantification assays

The assays were performed according to a literature procedure.^14,15^ Briefly, the peptide stock solutions were freshly prepared in Milli-Q water (for oxidized peptides) or 0.01 M HCl (for reduced peptides). The concentration of each stock solution was verified using the calculated extinction coefficients (Table S2†). The samples were prepared by diluting an aliquot of each peptide stock solution with Ar-purged assay buffer and adding a 42 μL aliquot of 2,2′-dithiodipyridine (DTDP) as previously described.^15^ The final peptide concentrations were 2–5 μM or 6–7 μM for the reduced proHD6 and oxidized HD6/proHD6, respectively. The resulting solutions were incubated at room temperature for at least 15 min and the absorbance at 341 nm was recorded. The number of free thiol residues in each peptide sample was determined by using a reduced glutathione (GSH) standard curve. These assays were conducted with at least two independently prepared and purified samples of each peptide and in three independent trials. The resulting averages (±SDM) are reported.

## References

[cit1] Turner J. R. (2009). Nat. Rev. Immunol..

[cit2] Zhao L., Lu W. (2014). Curr. Opin. Hematol..

[cit3] Ouellette A. J. (2010). Curr. Opin. Gastroenterol..

[cit4] Bevins C. L., Salzman N. H. (2011). Nat. Rev. Microbiol..

[cit5] Clevers H. C., Bevins C. L. (2013). Annu. Rev. Physiol..

[cit6] Jones D. E., Bevins C. L. (1992). J. Biol. Chem..

[cit7] Jones D. E., Bevins C. L. (1993). FEBS Lett..

[cit8] Mallow E. B., Harris A., Salzman N., Russell J. P., DeBerardinis R. J., Ruchelli E., Bevins C. L. (1996). J. Biol. Chem..

[cit9] Porter E. M., Bevins C. L., Ghosh D., Ganz T. (2002). Cell. Mol. Life Sci..

[cit10] Lehrer R. I., Lu W. (2012). Immunol. Rev..

[cit11] Ouellette A. J. (2011). Cell. Mol. Life Sci..

[cit12] Szyk A., Wu Z., Tucker K., Yang D., Lu W., Lubkowski J. (2006). Protein Sci..

[cit13] Maemoto A., Qu X., Rosengren K. J., Tanabe H., Henschen-Edman A., Craik D. J., Ouellette A. J. (2004). J. Biol. Chem..

[cit14] Wanniarachchi Y. A., Kaczmarek P., Wan A., Nolan E. M. (2011). Biochemistry.

[cit15] Chairatana P., Nolan E. M. (2014). J. Am. Chem. Soc..

[cit16] Ericksen B., Wu Z., Lu W., Lehrer R. I. (2005). Antimicrob. Agents Chemother..

[cit17] Chu H., Pazgier M., Jung G., Nuccio S.-P., Castillo P. A., de Jong M. F., Winter M. G., Winter S. E., Wehkamp J., Shen B., Salzman N. H., Underwood M. A., Tsolis R. M., Young G. M., Lu W., Lehrer R. I., Bäumler A. J., Bevins C. L. (2012). Science.

[cit18] Schroeder B. O., Ehmann D., Precht J. C., Castillo P. A., Küchler R., Berger J., Schaller M., Stange E. F., Wehkamp J. (2015). Mucosal Immunol..

[cit19] Porter E. M., Poles M. A., Lee J. S., Naitoh J., Bevins C. L., Ganz T. (1998). FEBS Lett..

[cit20] Rice W. G., Ganz T., Kinkade Jr J. M., Selsted M. E., Lehrer R. I., Parmley R. T. (1987). Blood.

[cit21] Valore E. V., Ganz T. (1992). Blood.

[cit22] Faurschou M., Sørensen O. E., Johnsen A. H., Askaa J., Borregaard N. (2002). Biochim. Biophys. Acta, Mol. Cell Res..

[cit23] Faurschou M., Kamp S., Cowland J. B., Udby L., Johnsen A. H., Calafat J., Winther H., Borregaard N. (2005). J. Leukocyte Biol..

[cit24] Ghosh D., Porter E., Shen B., Lee S. K., Wilk D., Drazba J., Yadav S. P., Crabb J. W., Ganz T., Bevins C. L. (2002). Nature.

[cit25] Wilson C. L., Ouellette A. J., Satchell D. P., Ayabe T., López-Boado Y. S., Stratman J. L., Hultgren S. J., Matrisian L. M., Parks W. C. (1999). Science.

[cit26] Ayabe T., Satchell D. P., Pesendorfer P., Tanabe H., Wilson C. L., Hagen S. J., Ouellette A. J. (2002). J. Biol. Chem..

[cit27] Bohe M., Borgström A., Lindström C., Ohlsson K. (1986). J. Clin. Pathol..

[cit28] Porter E. M., Liu L., Oren A., Anton P. A., Ganz T. (1997). Infect. Immun..

[cit29] Philo J. S. (2006). Anal. Biochem..

[cit30] Lebowitz J., Lewis M. S., Schuck P. (2009). Protein Sci..

[cit31] Vistica J., Dam J., Balbo A., Yikilmaz E., Mariuzza R. A., Rouault T. A., Schuck P. (2004). Anal. Biochem..

[cit32] Molmenti E. P., Perlmutter D. H., Rubin D. C. (1993). J. Clin. Invest..

[cit33] Glenthøj A., Glenthøj A. J., Borregaard N. (2013). Eur. J. Clin. Invest..

[cit34] Glenthøj A., Nickles K., Cowland J., Borregaard N. (2015). PLoS One.

[cit35] Cunliffe R. N., Rose F. R. A. J., Keyte J., Abberley L., Chan W. C., Mahida Y. R. (2001). Gut.

[cit36] Lichtenberger L. M. (1995). Annu. Rev. Physiol..

[cit37] Eddie lp W. K., Takahashi K., Ezekowitz R. A., Stuart L. M. (2009). Immunol. Rev..

[cit38] Williams R. C., Gibbons R. J. (1972). Science.

[cit39] Mantis N. J., Rol N., Corthésy B. (2011). Mucosal Immunol..

[cit40] Roche A. M., Richard A. L., Rahkola J. T., Janoff E. N., Weiser J. N. (2015). Mucosal Immunol..

[cit41] Zou G., de Leeuw E., Lubkowski J., Lu W. (2008). J. Mol. Biol..

[cit42] Chen Z., Koelsch G., Han H.-P., Wang X.-J., Lin X.-L., Hartsuck J. A., Tang J. (1991). J. Biol. Chem..

[cit43] Harris D. A., Huber M. T., van Dijken P., Shyng S.-L., Chait B. T., Wang R. (1993). Biochemistry.

[cit44] Sisodia S. S. (1992). Proc. Natl. Acad. Sci. U. S. A..

[cit45] Vassar R., Bennett B. D., Babu-Khan S., Kahn S., Mendiaz E. A., Denis P., Teplow D. B., Ross S., Amarante P., Loeloff R., Luo Y., Fisher S., Fuller J., Edenson S., Lile J., Jarosinski M. A., Biere A. L., Curran E., Burgess T., Louis J.-C., Collins F., Treanor J., Rogers G., Citron M. (1999). Science.

[cit46] Chiti F., Dobson C. M. (2006). Annu. Rev. Biochem..

[cit47] Jucker M., Walker L. C. (2013). Nature.

[cit48] Wehkamp J., Salzman N. H., Porter E., Nuding S., Weichenthal M., Petras R. E., Shen B., Schaeffeler E., Schwab M., Linzmeier R., Feathers R. W., Chu H., Lima H., Fellermann K., Ganz T., Stange E. F., Bevins C. L. (2005). Proc. Natl. Acad. Sci. U. S. A..

[cit49] Wu Z., Ericksen B., Tucker K., Lubkowski J., Lu W. (2004). J. Pept. Res..

[cit50] Tang P., Foubister V., Pucciarelli M. G., Finlay B. B. (1993). J. Microbiol. Methods.

[cit51] Johnson V. L., Ko S. C. W., Holmstrom T. H., Eriksson J. E., Chow S. C. (2000). J. Cell Sci..

[cit52] Wommack A. J., Robson S. A., Wanniarachchi Y. A., Wan A., Turner C. J., Wagner G., Nolan E. M. (2012). Biochemistry.

[cit53] Laue M., Shah B. D., Ridgeway T. M. and Pelletier S. L., in Analytical Ultracentrifugation in Biochemistry and Polymer Science, ed. S. Harding and A. Rowe, Royal Society of Chemistry, 1992, pp. 90–125.

[cit54] Philo J. S. (2000). Anal. Biochem..

