# Supplementary material for: Proteolysis triggers self-assembly and unmasks innate immune function of a human α-defensin peptide
Source: Chem Sci. 2015 Dec 10;7(3):1738–52. doi: 10.1039/c5sc04194e (PMC4827351; doi:10.1039/c5sc04194e)
Supplement: Supplementary file 1 [file SC-007-C5SC04194E-s001.pdf]

Electronic Supplementary Material (ESI) for Chemical Science.
This journal is © The Royal Society of Chemistry 2015

Supplementary Information for

Proteolysis Triggers Self-Assembly and Unmasks Innate Immune Function of a Human α-Defensin Peptide

Phoom Chairatana[1], Hiutung Chu[2,‡], Patricia A. Castillo[2,‡], Bo Shen[3], Charles L. Bevins[2], and Elizabeth M. Nolan[1,*]

[1]Department of Chemistry, Massachusetts Institute of Technology, Cambridge, MA 02139, USA

[2]Department of Microbiology and Immunology, University of California Davis School of Medicine, Davis, CA 95616, USA

[3]Department of General Internal Medicine and Gastroenterology and Hepatology, The Cleveland Clinic Foundation, Cleveland, OH 44195, USA

‡These authors contributed equally.

*Corresponding author: lnolan@mit.edu

Phone: 617-452-2495

Fax: 617-324-0505

This Supplementary Information section includes:

Supplementary Tables.. S3

Table S1. Amino acid sequences…………………………………..........………………….. S3

Table S2. Characterization of HD6 and proHD6..…………. S3

Table S3. Calculated sedimentation coefficients for proHD6.....................……………...... S4

Table S4. Sedimentation velocity results for proHD6.........................…………………..... S5

Table S5. Sedimentation equilibrium results for proHD6........................……….......... S6

Supplementary Figures.. S7

Figure S1. Characterization of HD6 in ileal fluid.. S7

Figure S2. Working model of HD6 maturation... S8

Figure S3. SDS-PAGE and HPLC traces of proHD6.. S8

Figure S4. Antibacterial activity assays of proHD6.. S9

Figure S5. *In vitro* trypsin-catalyzed degradation of proHD6.. S9

Figure S6. Characterization of products from trypsinized proHD6.................................. S10

Figure S7. TEM of trypsinized proHD6 in Tris-maleate buffer... S11

Figure S8. TEM of trypsinized proHD6 at different time points....................................... S11

Figures S9-S12. SEM of bacterial agglutination by trypsinized proHD6.......................... S12

Figure S13. Cytotoxicity studies of proHD6 and HD6 against T84 cells.......................... S16

Figure S14. Sedimentation velocity analysis of proHD6.. S17

Figure S15. Sedimentation equilibrium analysis of proHD6.. S17

Supplementary References.. S18

Table S1. Amino acid sequences.

Peptide/Protein	Amino Acid Sequence
preproHD6	MRTLTILTAVLLVALQAKAEPLQAEDDPLQAKAYEADAQEQRGANDQDFAVS FAEDASSSLRALGSTRAFTCHCRRSCYSTEYSYGTCTVMGINHRFCCL
proHD6	EPLQAEDDPLQAKAYEADAQEQRGANDQDFAVSFAEDASSSLRALGSTRAF TCHCRRSCYSTEYSYGTCTVMGINHRFCCL
HD6	AFTCHCRRSCYSTEYSYGTCTVMGINHRFCCL
His_6-SUMO-proHD6	GSSHHHHHHGSGLVPRGSASMSDSEVNQEAKPEVKPEVKPETHINLKVSDG SSEIFFKIKKTTPLRRLMEAFAKRQGKEMDSLRFLYDGIRIQADQTPEDLDME DNDIIEAHREQIGGEPLQAEDDPLQAKAYEADAQEQRGANDQDFAVSFAEDA SSSLRALGSTRAFTCHCRRSCYSTEYSYGTCTVMGINHRFCCL

Table S2. Characterization of peptides and protein employed in this work.

Peptide/Protein[a]	Retention Time (min)[b]	Free Thiol[c]	Calculated *m/z*	Observed *m/z*	Yield (mg/L culture)	ε_{280} ($M^{-1}cm^{-1}$)[d]
HD6	16.9	0.23 ± 0.08	3708.2	3707.4	1.9	4845
$proHD6_{red}$	22.4	6.31 ± 0.46	8966.7	8966.9	0.50	5960
proHD6	21.6	0.15 ± 0.20	8960.7	8961.2	0.20	6335
His_6-SUMO-proHD6	n.d.[e]	n.d.[e]	22227	22228	20	7825

[a] See Table S1 for amino acid sequences. HD6 and proHD6 are the oxidatively folded peptides with three S—S bonds. [b] Retention times determined by using analytical RP-HPLC on a C18 column and a gradient of 10–60% B over 30 min at 1 mL/min. [c] Free thiol content determined by using the DTDP assay (mean ± SDM, n ≥ 3). [d] Extinction coefficients at 280 nm were calculated by using the on-line resource ExPASy ProtParam. [e] n.d. = not determined.

Table S3. Sedimentation coefficient calculations using the Svedberg-Stokes equation.[1]

Species	S_{sphere} (S)[a]	S_{sphere} (S)[b]	S_{sphere} (S)[c]	Partial Specific Volume (mL/g)
proHD6 monomer	1.743	1.746	1.748	0.7011
proHD6 dimer	2.767	2.771	2.775	0.7011

[a] Buffer conditions: 10 mM sodium phosphate buffer, pH 7.4, of which a solvent density (ρ) of 0.99967 g/mL and a solvent viscosity (η) of 1.0061 cP at 20 °C. Viscosity units are in centipoise (cP) (1 poise = 1 $g \cdot cm^{-1} \cdot s^{-1}$). Sedimentation coefficients are in svedbergs (1 svedberg = 100 fs = 1×10^{-13} s). [b] Buffer conditions: 10 mM HEPES, pH 7.4, of which a solvent density (ρ) of 0.99901 g/mL, and a solvent viscosity (η) of 1.0104 cP at 20 °C. [c] Buffer conditions: 10 mM Tris-HCl, pH 7.4, of which a solvent density (ρ) of 0.99851 g/mL, and a solvent viscosity (η) of 1.0037 cP at 20 °C. The solvent densities, viscosities, and the partial specific volume values of proHD6 monomer and dimer were predicted by SEDNTERP.

Table S4. Calculated sedimentation coefficients of proHD6.[a]

Concentration (μM)	Buffer (10 mM)	pH	$s_{20,w}$ (S)	*D* (F)	MW (kDa)	Partial Specific Volume (mL/g)
30	NaP	7.4	1.727	9.83	14.3	0.7011
30	NaP	7.4	1.798	11.5	12.8	0.7011
30	NaP	7.4	1.733	9.73	14.5	0.7011
50	NaP	7.4	1.844	10.4	14.4	0.7011
50	NaP	7.4	1.865	10.3	14.8	0.7011
100	NaP	7.4	1.843	13.8	10.9	0.7011
120	NaP	7.4	1.769	9.16	15.7	0.7011
140	NaP	7.4	1.751	9.47	15.0	0.7011
50	HEPES	7.4	1.763	9.94	14.4	0.7011
50	Tris-HCl	7.4	1.783	8.97	16.1	0.7011

[a] All samples were prepared in the indicated buffer; NaP is sodium phosphate. The temperature was 20 °C. Data were obtained by analysis with the dc/dt method implemented in DCDT+ using 22-28 scans with 40 kDa diffusion broadening maximum. Sedimentation coefficients are $s_{20,w}$ values, adjusted with a solvent density (ρ) of 0.99967 g/mL and a solvent viscosity (η) of 1.0061 cP. Viscosity units are in centipoise (cP) (1 poise = 1 $g{\bullet}cm^{-1}{\bullet}s^{-1}$). Sedimentation coefficients are in svedbergs (1 svedberg = 100 fs = 1 x 10^{-13} s). Diffusion coefficients correspond to the best-fit molecular mass in Fick units (1 Fick = 1 x 10^{-7} cm^2/s). HEPES has a solvent density (ρ) of 0.99901 g/mL and a solvent viscosity (η) of 1.0104 cP at 20 °C. Tris-HCl has a solvent density (ρ) of 0.99851 g/mL and a solvent viscosity (η) of 1.0037 cP at 20 °C. The solvent densities, viscosities, and the partial specific volume values of proHD6 monomer and dimer were predicted by SEDNTERP.

Table S5. Molecular weight fitting of SE data for proHD6 by using SEDPHAT.[a]

Sample	MW (Da)	95% Confidence Interval[b]	Standard Deviation[b]	Global Reduced Chi-Squared Value	Critical Chi-Squared Value[c]
1	15176	14719-15693 Da	± 250 Da	0.2344	0.3942
2	15351	14745-15884 Da	± 295 Da	0.4953	0.8396
3	15549	15059-15946 Da	± 230 Da	0.3472	0.5717

[a] All samples were in 10 mM sodium phosphate, pH 7.4. Global fitting used partial specific volume ($\bar{v}$) at 20 °C, a solvent density (ρ) of 0.99967 g/mL, and a solvent viscosity (η) of 1.0061 cP. Viscosity units are in centipoise (cP) (1 poise = 1 g•cm^{-1}•s^{-1}). [b] Calculated from 1000 iterations of Monte-Carlo analysis. [c] Calculated at a 95% confidence level.

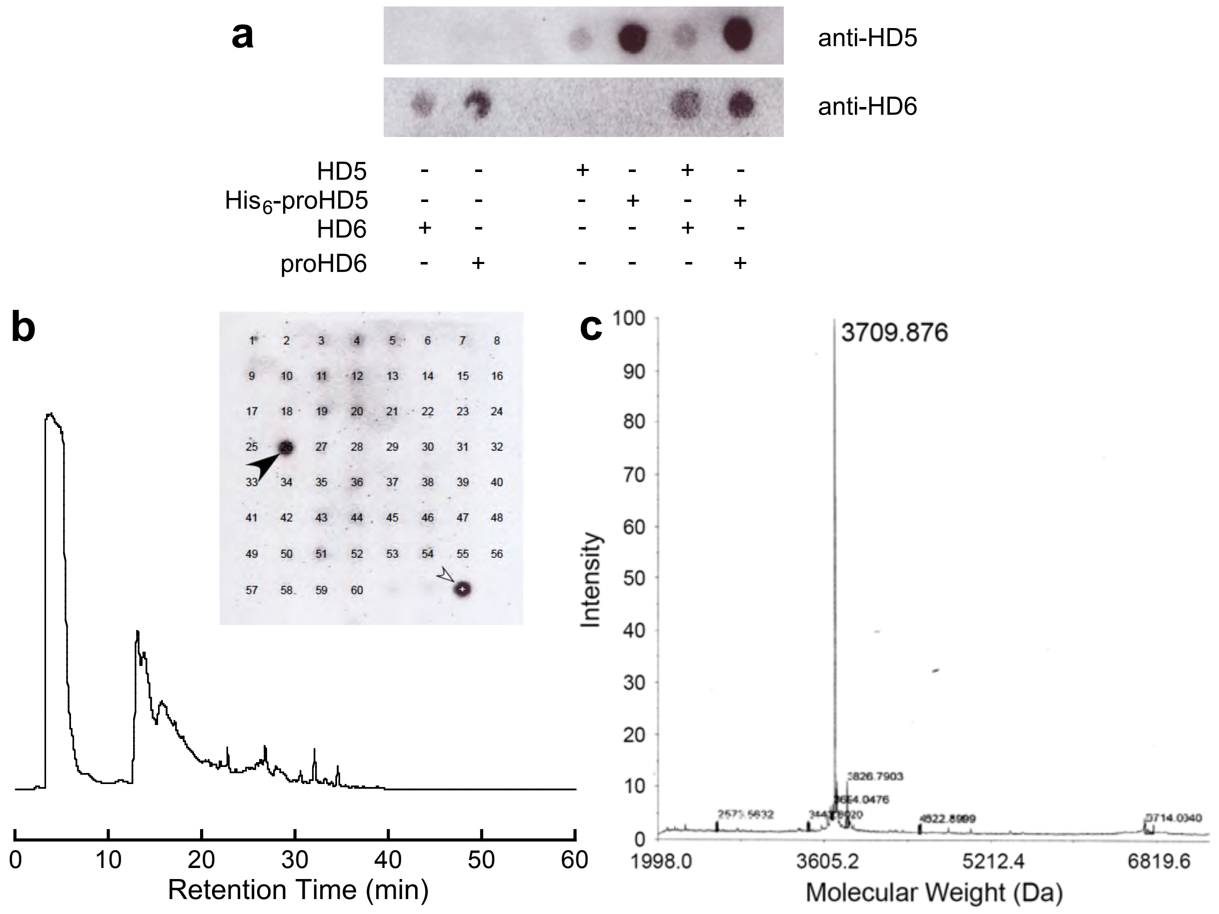

Figure S1. Characterization of HD6 in ileal fluid. (a) A dot blot showing specificity of the antibodies employed in this work. HD5 (0.14 nmol), HD6 (0.14 nmol), His_6-proHD5 (0.16 nmol), and proHD6 (0.16 nmol) in Milli-Q water were spotted directly onto an Immobilon PSQ PVDF membrane. (b) Analysis of ileal lumen fluid aspirate. Ileal lumenal fluid, obtained by endoscopy aspiration, was acidified (20% acetic acid) clarified by centrifugation, and fractionated by analytical C18 RP-HPLC using a gradient of 5–62% acetonitrile over 60 min at 1 mL/min (280 nm absorbance). A 20-μL aliquot of each 1-mL fraction was analyzed for HD6 immunoreactivity by dot blot analysis (inset). A second aliquot (2 μL) was screened by MALDI-TOF MS analysis, to identify masses that potentially corresponded to HD6 isoforms. A single fraction (26, black arrow head) was positive for HD6. Recombinant mature HD6 (residues 69-100, 1 μg) was spotted as a positive control (open arrowhead). (c) MALDI-TOF MS analysis of fraction 26.

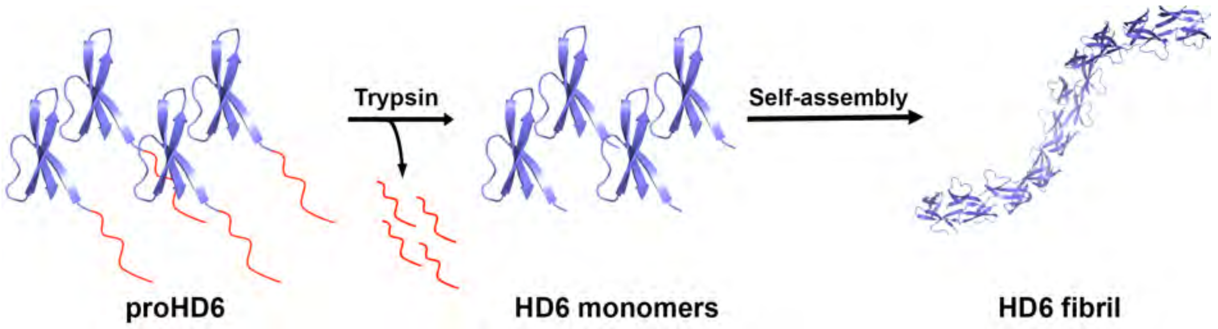

Figure S2. Model for HD6 maturation and self-assembly. The proposed model where proteolysis of proHD6 by trypsin generates the HD6 monomer and triggers its self-assembly into fibrils (PDB ID: 1ZMQ).[2]

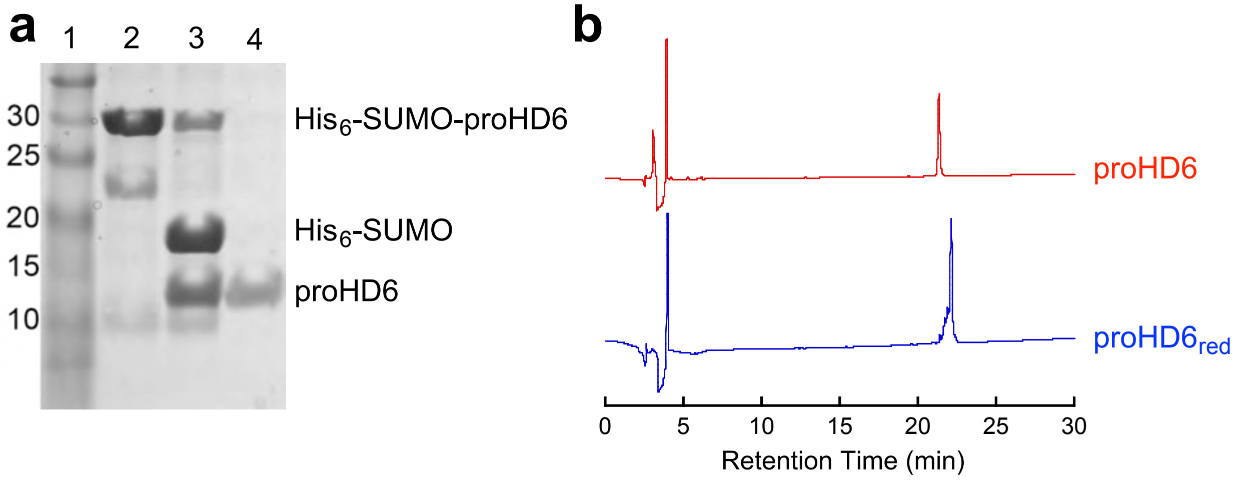

Figure S3. Preparation and purification of proHD6. (a) SDS-PAGE (15% Tris-HCl gel) of samples from a representative preparation of proHD6. His_6-SUMO-proHD6 is 22.2 kDa, His_6-SUMO is 13.3 kDa, and proHD6 is 8.96 kDa. Lane 1: P7711S pre-stained gel ladder (New England Biolabs). Lane 2: purified His_6-SUMO-proHD6 from Ni-NTA chromatography. Lane 3: His_6-SUMO-proHD6 after incubated with Ulp1 (1% m/m) for 2 h. Lane 4: proHD6. (b) Analytical HPLC traces of purified proHD6 (variable sample concentrations). Absorbance at 220 nm was monitored with a reference wavelength of 500 nm. Method: 10-60% B over 30 min at 1 mL/min.

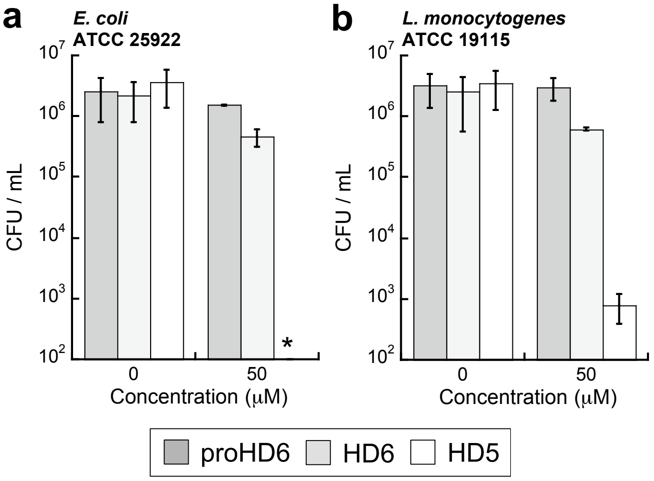

Figure S4. Antibacterial activity assays against *E. coli* ATCC 25922 and *L. monocytogenes* ATCC 19115. The bacteria were treated with 50 μM of each peptide for 1 h at 37 °C in 10 mM sodium phosphate buffer containing 1% v/v TSB (mean ± SDM, n = 3). An asterisk indicates no colony formation.

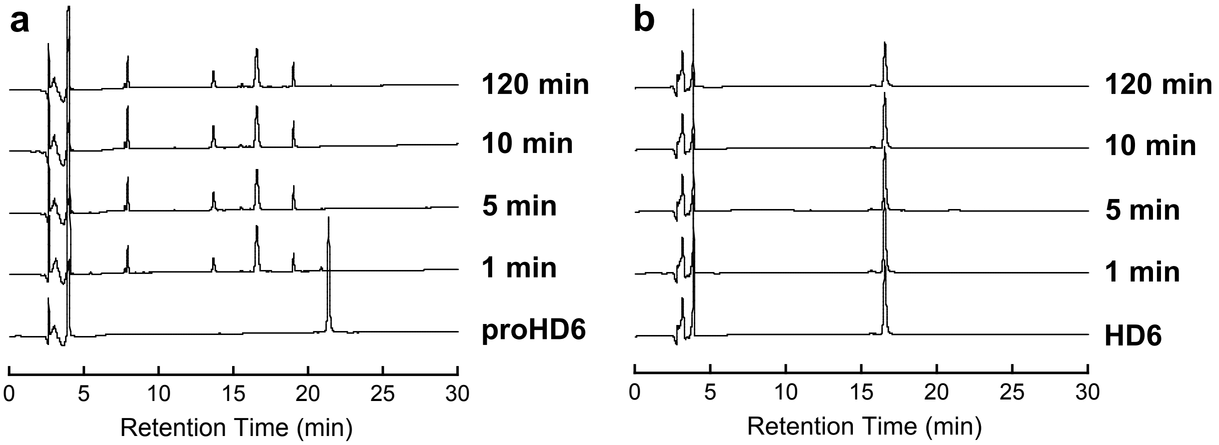

Figure S5. *In vitro* trypsin-catalyzed proteolysis of proHD6. Analytical HPLC traces of trypsin-treated (a) proHD6 or (b) mature HD6 (30 μM x 80 μL) at indicated time points. Absorbance at 220 nm was monitored with a reference wavelength of 500 nm. Method: 10–60% B over 30 min at 1 mL/min.

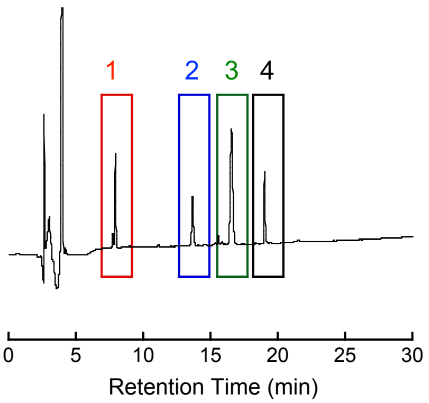

EPLQAEDDPL QAKAYEADAQ EQRGANDQDF AVSFAEDASS
SLRALGSTRA FTCHCRRSCY STEYSYGTCT VMGINHRFCC L

Fraction	Sequence	Retention Time (min)	Calculated *m/z*	Observed *m/z*
1	AYEADQAEQR	7.90	1180.5	1180.5
2	EPLQAEDDPLQAK	13.6	1452.7	1452.7
3	AFTCHCRRSCYSTEYSYGTC TVMGINHRFCCL	16.5	3705.5	3705.5
4	GANDQDFAVSFAEDASSSLR	19.0	2086.9	2087.0
-	ALGSTR	n.d.[a]	603.33	n.d.[a]

[a]n.d. = not detected.

Figure S6. Characterization of products from trypsin-catalyzed proteolysis of proHD6. Analytical HPLC trace of proHD6 (30 μM x 80 μL) incubated with 0.4 μM trypsin after 2 h (a 1:100 trypsin:proHD6 mass ratio in 100 mM Tris-HCl, 20 mM $CaCl_2$, pH 8.0) The starting polypeptide proHD6, which elutes at 21.6 min, was completely hydrolysed. Absorbance at 220 nm was monitored with a reference wavelength of 500 nm. Method: 10–60% B over 30 min at 1 mL/min. The HPLC retention times, *m/z* values, and amino acid sequences of the four isolated products are summarized in the table. The amino acid sequence for HD6 is given in Figure 1 and Table S1.

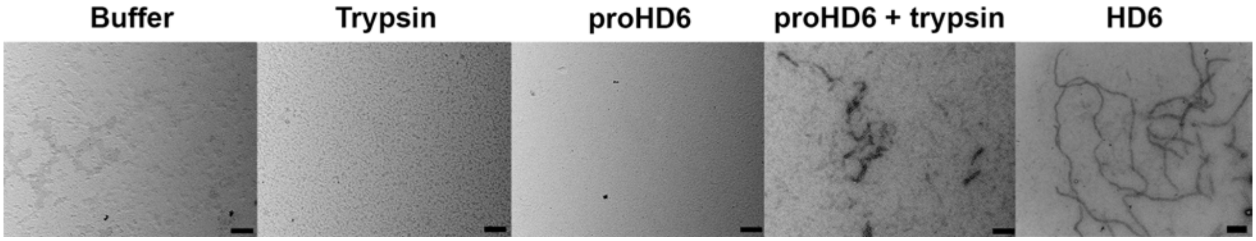

Figure S7. TEM analysis of trypsin-catalyzed proteolysis of proHD6 in Tris-maleate buffer. Transmission electron micrographs of 10 mM Tris-maleate pH 6.4 (control), 0.4 μM trypsin (control), 20 μM proHD6 in the absence and presence of 0.4 μM trypsin, and 20 μM HD6. All samples were incubated at room temperature for 1 h. Scale bar = 200 μm.

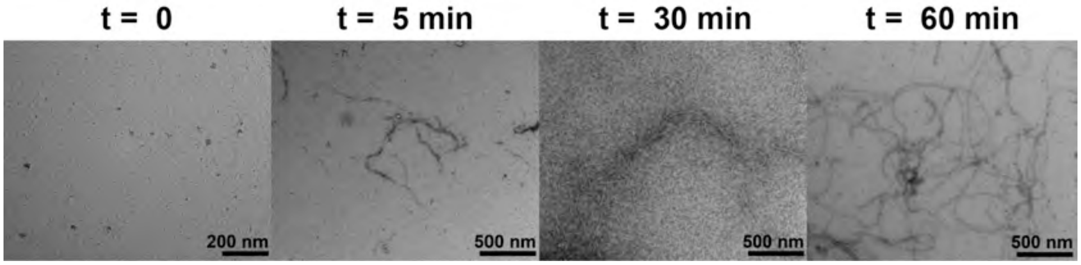

Figure S8. Time course of trypsin-catalyzed proteolysis of proHD6 monitored by TEM. Transmission electron micrographs of 20 μM proHD6 after treatment with 0.4 μM trypsin at room temperature for the indicated time. All the samples were in 10 mM sodium phosphate buffer, pH 7.4.

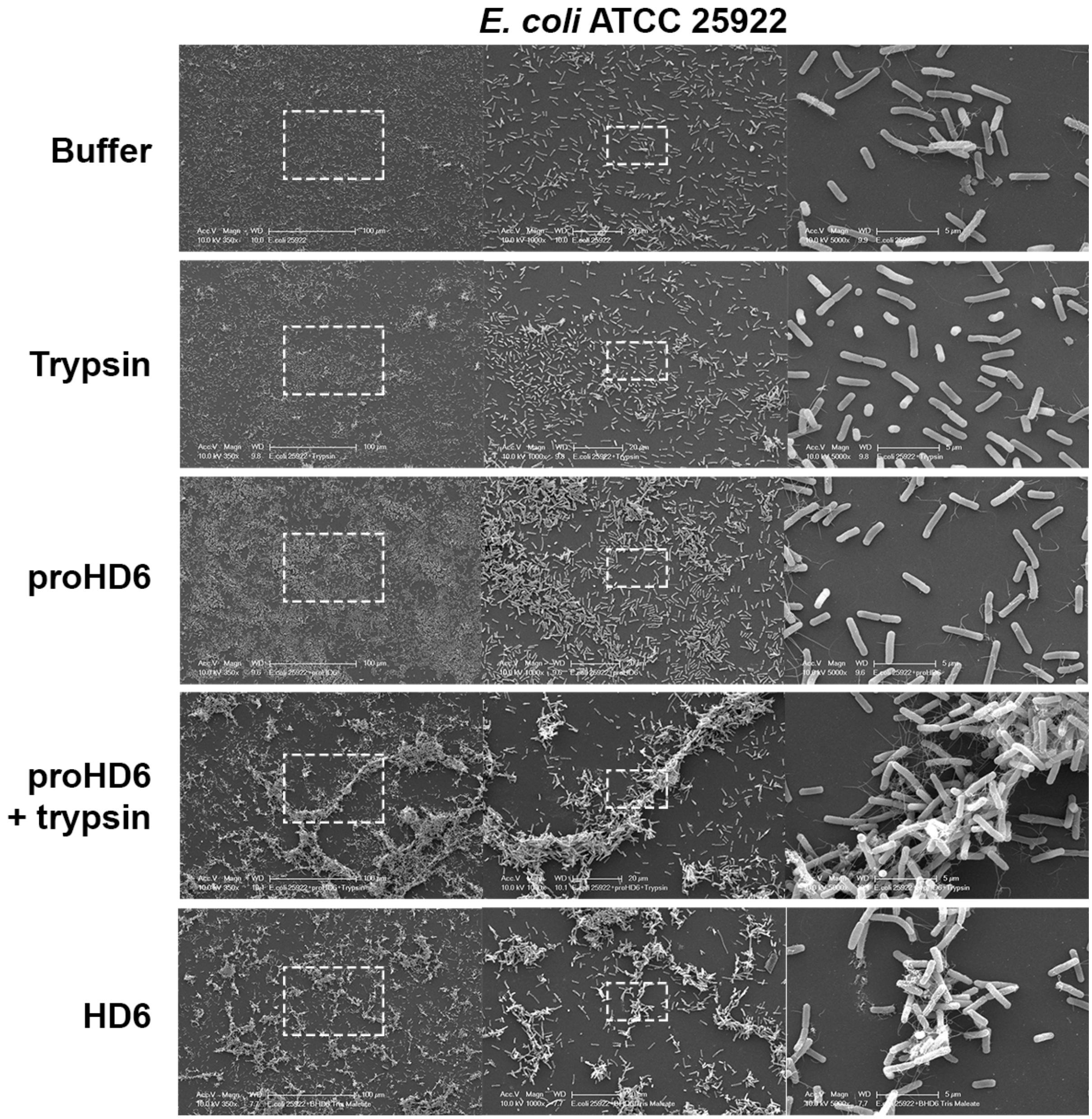

Figure S9. SEM analysis of *E. coli* ATCC 25922 agglutination by trypsin-cleaved proHD6. Scanning electron micrographs of *E. coli* ATCC 25922 treated with buffer only, 0.4 μM APMSF-inactivated trypsin, 3 μM proHD6, 3 μM trypsin-cleaved proHD6, or 3 μM HD6 (50 mM Tris-maleate pH 6.4). Trypsin-cleaved proHD6 was prepared prior to incubation with the bacteria and the residual enzymatic activity was inhibited by APMSF as described in the experimental section. Scale bar = 100 μm (left column), 20 μm (middle column), and 4 μm (right column). Dotted rectangles indicate field of view shown at higher magnifications.

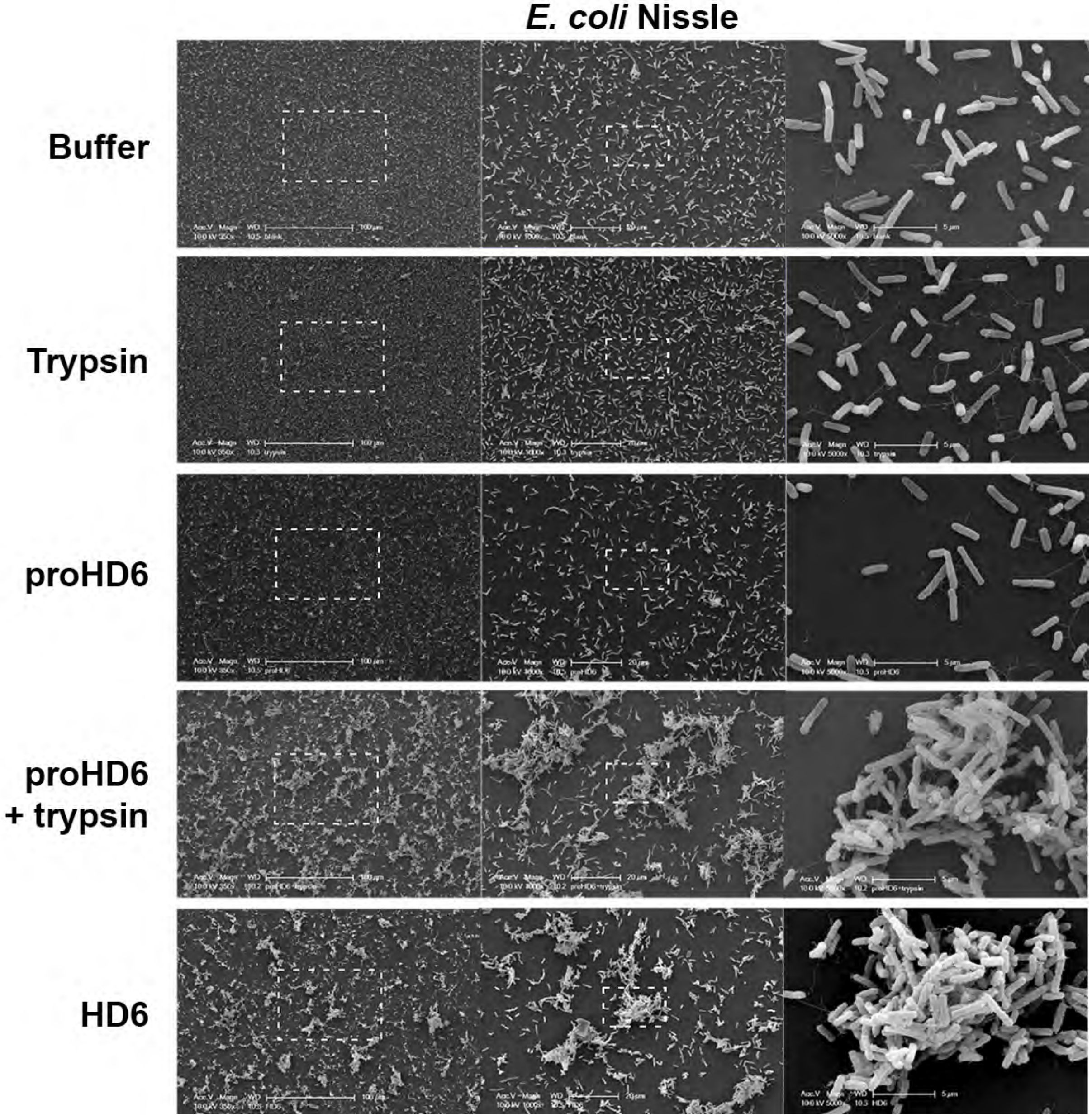

Figure S10. SEM analysis of *E. coli* Nissle agglutination by trypsin-cleaved proHD6. Scanning electron micrographs of *E. coli* Nissle treated with buffer only, 0.4 μM APMSF-inactivated trypsin, 3 μM proHD6, 3 μM trypsin-cleaved proHD6, or 3 μM HD6 (50 mM Tris-maleate pH 6.4). Trypsin-cleaved proHD6 was prepared prior to incubation with the bacteria and the residual enzymatic activity was inhibited by APMSF as described in the experimental section. Scale bar = 100 μm (left column), 20 μm (middle column), and 4 μm (right column). Dotted rectangles indicate field of view shown at higher magnifications.

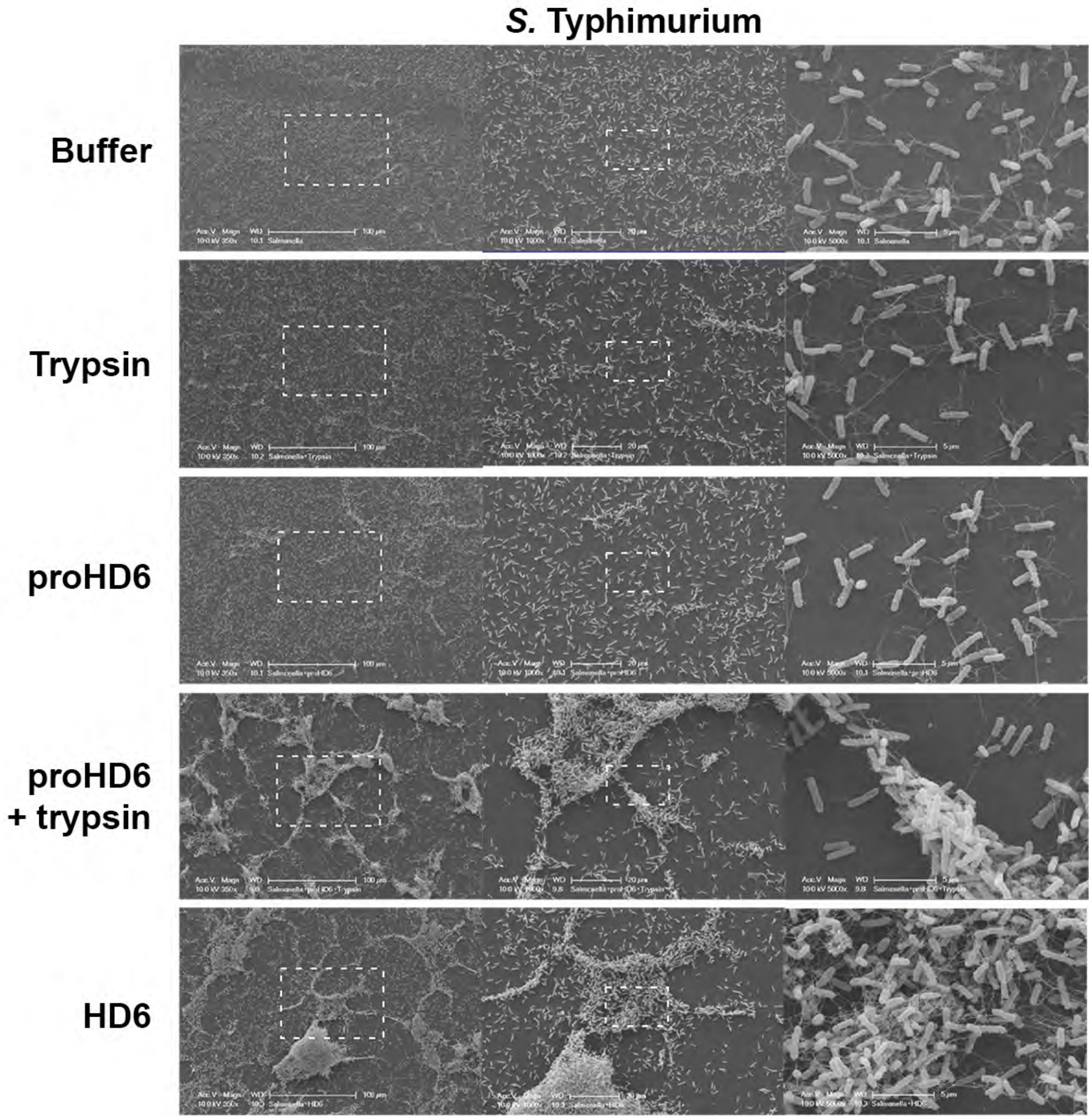

Figure S11. SEM analysis of *Salmonella enterica* serovar Typhimurium agglutination by trypsin-cleaved proHD6. Scanning electron micrographs of *S.* Typhimurium treated with buffer only, 0.4 μM APMSF-inactivated trypsin, 3 μM proHD6, 3 μM trypsin-cleaved proHD6, or 3 μM HD6 (50 mM Tris-maleate, pH 6.4). Trypsin-cleaved proHD6 was prepared prior to incubation with the bacteria and the residual enzymatic activity was inhibited by APMSF as described in the experimental section. Scale bar = 100 μm (left column), 20 μm (middle column), and 4 μm (right column). Dotted rectangles indicate field of view shown at higher magnifications.

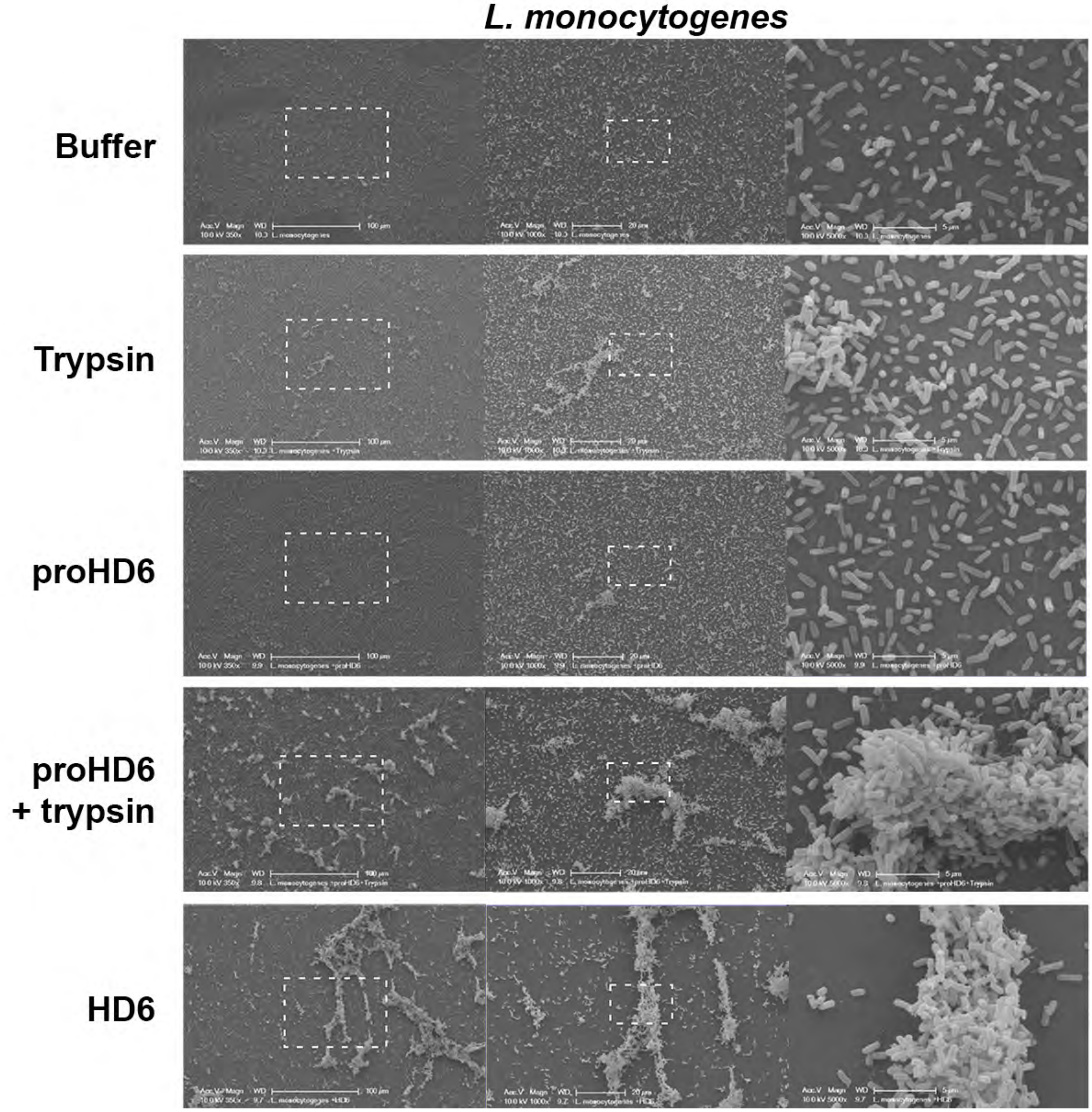

Figure S12. SEM analysis of *Listeria* agglutination by trypsin-cleaved proHD6. Scanning electron micrographs of *L. monocytogenes* treated with buffer only, 0.4 μM APMSF-inactivated trypsin, 3 μM proHD6, 3 μM trypsin-cleaved proHD6, or 3 μM HD6 (50 mM Tris-maleate pH 6.4). Trypsin-cleaved proHD6 was prepared prior to incubation with the bacteria and the residual enzymatic activity was inhibited by APMSF as described in the experimental section. Scale bar = 100 μm (left column), 20 μm (middle column), and 4 μm (right column). Dotted rectangles indicate field of view shown at higher magnifications.

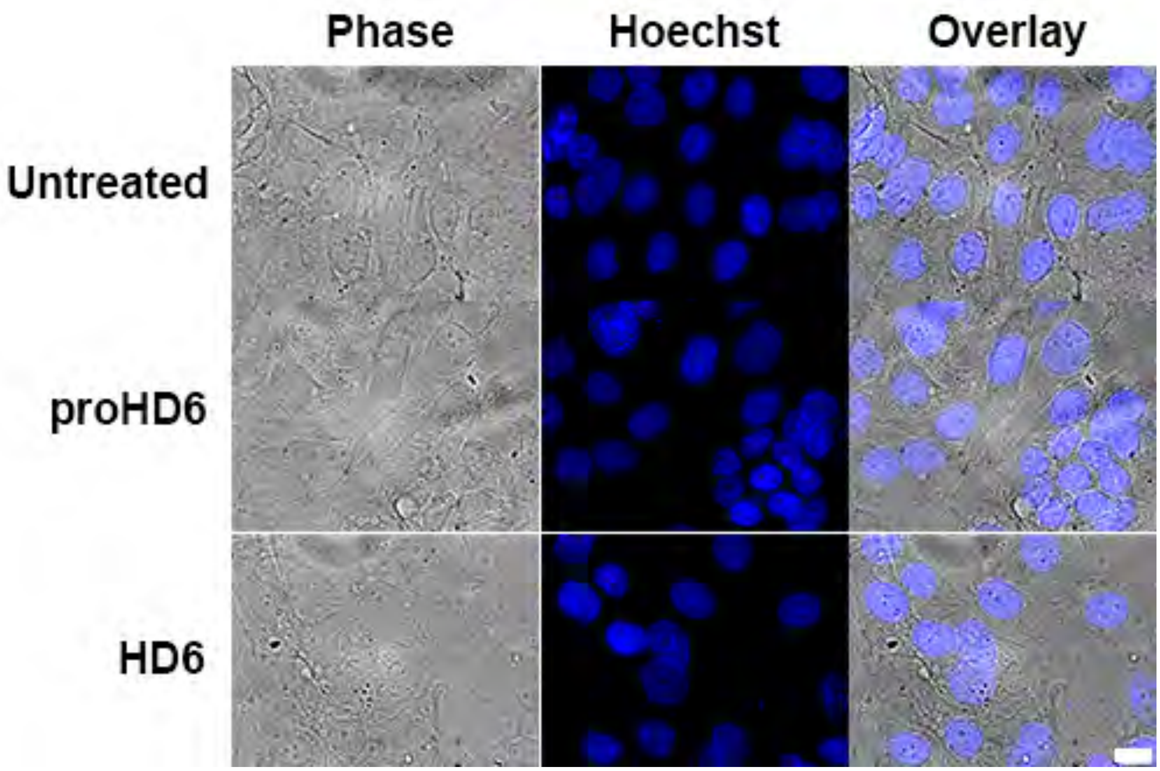

Figure S13. Cytotoxicity studies of proHD6 and mature HD6 against human intestinal epithelial cells (T84 cells). Representative images of T84 cells after incubated with 20 μM peptides (t = 1.5 h, T = 37 °C, 5% CO_2). Excitation wavelength = 360 ± 20 nm. A scale bar = 10 μm.

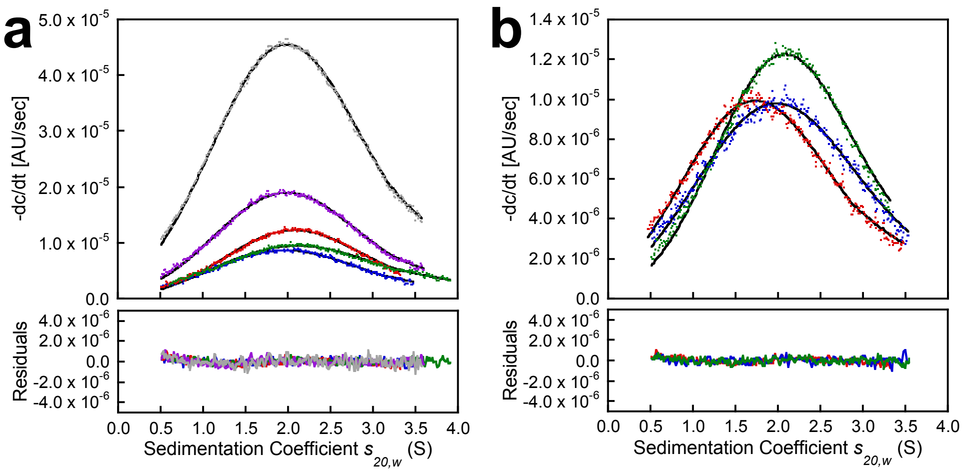

Figure S14. Sedimentation velocity analysis of proHD6 under different conditions. (a) Analytical ultracentrifugation of proHD6 at concentrations ranging from 30 μM to 140 μM in 10 mM sodium phosphate buffer, pH 7.4. (b) Analytical ultracentrifugation of proHD6 in 10 mM sodium phosphate pH 7.4 (red dots), HEPES pH 7.4 (blue dots), and Tris-HCl pH 7.4 (green dots). The colored dots are the −dc/dt data obtained from sedimentation velocity experiments (absorbance at 280 nm). The black lines are the single Gaussian fits obtained using DCDT+. The summary of the fits is provided in Table S4.

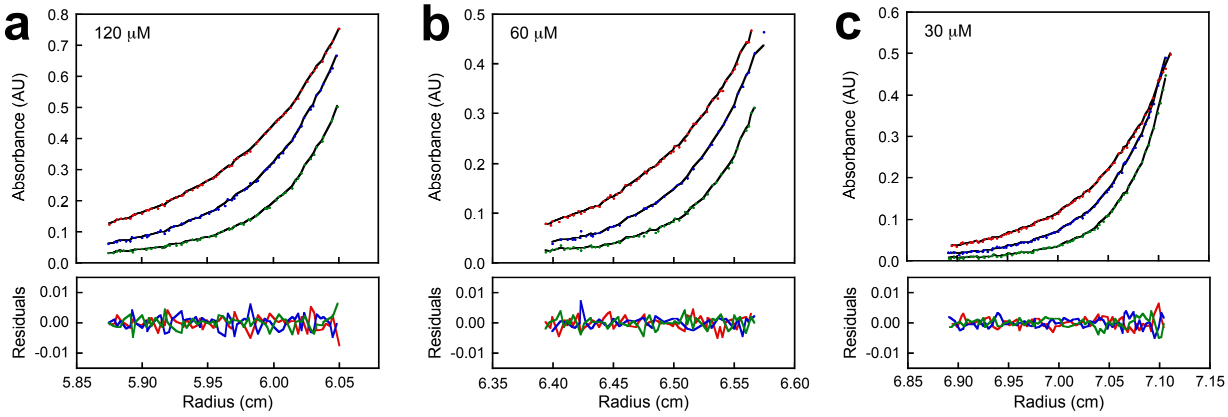

Figure S15. Sedimentation equilibrium analysis of proHD6. Representative sedimentation equilibrium profiles of proHD6 in 10 mM sodium phosphate buffer, pH 7.4 at high (a), medium (b), and low (c) concentrations. Best fits (black lines) of raw UV absorbance at 280 nm at rotor speeds of 30 000 (red), 36 000 (blue), and 42 000 (green) rpm. The fits and calculated molecular weights are summarized in Table S5.

Supplementary References

1. J. Lebowitz, M. S. Lewis and P. Schuck, *Protein Sci.*, 2002, **11**, 2067-2079.
2. A. Szyk, Z. Wu, K. Tucker, D. Yang, W. Lu and J. Lubkowski, *Prot. Sci.*, 2006, **15**, 2749-2760.
